# Bile Acids and the Gut–X Axis: TCM-Mediated Systemic Protection and Therapeutic Opportunities for Multi-Organ Diseases

**DOI:** 10.3390/metabo16060366

**Published:** 2026-05-28

**Authors:** Jialu He, Linjie Qin, Xian Sun

**Affiliations:** School of Integrated Chinese and Western Medicine, Nanjing University of Chinese Medicine, Nanjing 210023, China; hejialll@njucm.edu.cn (J.H.); qinlinjie@njucm.edu.cn (L.Q.)

**Keywords:** gut microbiota, bile acids, gut–X axis, host receptors, traditional Chinese medicine

## Abstract

The gut microbiota regulates host physiology and drives extraintestinal diseases through the gut–X axis. Bile acids (BAs) function as key mediators of this interorgan crosstalk by activating nuclear and membrane receptors (FXR, TGR5, PXR, VDR). Traditional Chinese Medicine (TCM) demonstrates efficacy across multiple organ systems through multi-component formulations. This narrative review synthesizes evidence from preclinical and clinical studies supporting that TCM exerts systemic protection via strategic modulation of the microbiota–BA–host receptor axis, which functions as a core regulatory circuit within a larger network of microbial metabolites. Mechanistically, representative TCM formulas remodel gut microbial ecology and reinforce intestinal barrier integrity, leading to optimized BA profiles. These favorable BA signatures engage tissue-specific receptor signaling to resolve inflammation, mitigate fibrosis, and restore metabolic homeostasis across the gut–heart, gut–kidney, gut–liver, gut–bone, and gut–endocrine axes. Support for this causal relationship is provided by microbiota depletion, fecal transplantation, and multi-omics studies, collectively suggesting that TCM’s benefits are microbiota-dependent and at least partially BA-mediated. Moreover, context-dependent modulation of BA receptors, such as differential regulation of FXR, enables TCM to achieve pathology-specific outcomes. Current evidence is derived predominantly from preclinical models, and clinical data remain lacking. Nonetheless, the microbiota–BA–organ axis thus provides a potential framework for understanding TCM’s systemic actions and establishes a molecular basis for developing microbiome-informed precision therapeutics. Future directions include patient stratification and precision intervention design inspired by TCM’s ecological modulation strategies.

## 1. Introduction

The gut microbiota (GM) constitutes an exceptionally diverse ecosystem containing trillions of microorganisms that fundamentally reshape our understanding of systemic disease pathogenesis by serving as a central regulator of host physiology [1,2]. This complex microbial community engages in dynamic bidirectional communication with the host through integrated metabolic and signaling networks [3]. Recent conceptual advances describe this crosstalk through the gut–X axis framework, illustrating how the GM profoundly influences distant organ systems and establishing the gut as a master regulator of systemic homeostasis [4,5,6,7].

The molecular foundation of this inter-organ communication resides primarily in the metabolic activities of GM. GM transform dietary constituents and host-derived compounds into a vast spectrum of bioactive metabolites that extend their influence beyond local intestinal effects to exert systemic regulatory actions. These microbial metabolites operate as crucial signaling molecules, endocrine factors, and metabolic regulators that coordinate physiological responses in distal tissues including the heart, kidneys, liver, joints, and endocrine organs [8,9,10,11,12]. The systemic impact of these gut-derived signals is modulated by the intestinal barrier’s selective permeability, which determines the communication between luminal contents and internal homeostasis [13].

The intestinal mucosal barrier represents a multilayered biological structure integrating physical, chemical, immunological, and microbial components that function synergistically to preserve intestinal homeostasis [14]. Its physical foundation is an intestinal epithelial monolayer, sealed by tight junction (TJ) proteins such as Occludin, Claudins, and Zonula occludens [15]. Selectively permeable structure facilitates nutrient absorption while restricting the ability of luminal microbiota to translocate and deliver potentially harmful metabolites [13,14]. However, compromise of this critical barrier function, a state often termed increased intestinal permeability or “leaky gut”, initiates multiple pathological cascades [16]. The mechanistic basis for this transition from homeostasis to disease lies in the loss of barrier integrity, which permits the translocation of luminal contents, including microbial components, viable bacteria, and microbial metabolites, into systemic circulation. This disruption has profound systemic consequences, primarily driven by the translocation of specific microbial products. Among these, bacterial lipopolysaccharide (LPS) has emerged as a key driver of systemic inflammation. The passive movement of LPS across the compromised intestinal barrier and its subsequent recognition by Toll-like receptor 4 (TLR4) represents a paradigmatic mechanism directly linking intestinal barrier dysfunction to sustained systemic inflammation [8,17]. Upon TLR4 activation, LPS triggers potent inflammatory responses primarily through the nuclear factor-κB (NF-κB) pathway, leading to the robust production of pro-inflammatory cytokines [18]. Notably, this inflammatory response operates as a self-amplifying loop. These cytokines not only perpetuate local intestinal inflammation but also simultaneously exert systemic effects including endothelial dysfunction, immune cell activation in distant organs, and disruption of metabolic homeostasis, thereby exacerbating both local and systemic pathology. Critically, these same inflammatory mediators further compromise intestinal barrier integrity by directly damaging TJ proteins, establishing a self-perpetuating cycle of barrier deterioration and escalating inflammation. The clinical relevance of this gut-derived systemic inflammation is profound and far-reaching. Its pathological consequences extend across multiple organ systems, contributing to the pathogenesis of diverse conditions including atherosclerosis, non-alcoholic fatty liver disease (NAFLD), chronic kidney disease (CKD), metabolic syndrome, and autoimmune disorders [4,5,6,7]. Thus, the intestinal barrier serves not merely as a physical partition but as a critical determinant of systemic health, with its dysfunction representing a common pathogenic pathway across multiple organ systems.

Beyond these pro-inflammatory pathways, the GM also generates metabolites with potent regulatory and protective functions. Among these, bile acids (BAs) occupy a uniquely central position due to their dual roles in lipid digestion and systemic signaling [19]. A metabolic pathway exemplifies well-characterized host–microbiota co-metabolism, wherein host synthesis and microbial modification are tightly coupled. Primary BAs synthesized from cholesterol in hepatocytes undergo conjugation with glycine or taurine before biliary secretion [20]. Following digestive functions, approximately 95% of BAs undergo enterohepatic reabsorption, while the remaining 5% reach the colon where gut bacteria transform them through enzymatic processes including deconjugation by bile salt hydrolases (BSHs), dehydroxylation, oxidation, and isomerization [21]. These microbial transformations generate diverse secondary BAs with distinct physicochemical and biological properties [22]. The functional significance of this metabolic diversification lies in the ability of BAs to act as multifaceted signaling molecules. They exert their effects through activation of multiple host receptors, including nuclear receptors such as farnesoid X receptor (FXR), pregnane X receptor (PXR), constitutive androstane receptor (CAR), and vitamin D receptor (VDR), as well as G protein-coupled receptors such as TGR5 [23,24]. Two receptors merit particular attention due to their broad regulatory roles. FXR, activated by both free and conjugated BAs, functions as a transcription factor regulating genes involved in BA synthesis, transport, and metabolism, playing a crucial role in BA homeostasis [25]. TGR5, activated by conjugated and unconjugated BAs, activates intracellular signaling cascades through cyclic AMP production, influencing processes including energy metabolism and inflammation [24]. The tissue distribution of these receptors further underscores the systemic reach of BA signaling. Expression has been documented in the liver, intestine, immune cells, adipose tissue, heart, kidney, skeletal muscle, and central nervous system [26,27,28]. Through these receptors, BAs regulate diverse physiological processes including glucose homeostasis, lipid metabolism, energy expenditure, thermogenesis, and intestinal barrier function [24]. Notably, BA signaling intersects directly with intestinal barrier function. FXR and TGR5 activation in intestinal epithelial cells upregulates expression of TJ proteins including ZO-1 and Occludin, enhancing barrier integrity and positioning BAs as potential counterregulatory agents against barrier disruption induced by inflammatory stimuli [29]. Furthermore, BA signaling modulates the gut immune system by influencing macrophage polarization, regulatory T cell differentiation, and cytokine production [30,31].

Thus, BAs function as multifaceted signaling molecules at the intersection of GM ecology and host systemic physiology, with the capacity to modulate intestinal barrier function, immune responses, and metabolic homeostasis [32]. This functional versatility raises a fundamental question regarding how these diverse signaling activities are coordinated within the broader context of host–microbiota interactions. The bidirectional relationship between BA metabolism and gut microbial composition creates a complex regulatory loop wherein GM shape the BA pool, which in turn influences microbial ecology and host physiology [33]. This interdependence positions the microbiota-BA axis as a central hub for gut–X axis communication, offering multiple points for therapeutic intervention. Building upon this mechanistic understanding, Traditional Chinese Medicine (TCM) emerges as a particularly relevant therapeutic paradigm. Clinically, TCM has demonstrated efficacy in treating extraintestinal diseases including cardiovascular disorders, CKD, and metabolic conditions, all of which are now understood to be closely linked to GM dysbiosis and intestinal barrier dysfunction [34,35,36]. Mechanistically, growing research supports that TCM formulations reshape gut microbial ecology, enhance intestinal barrier integrity, and reprogram gut microbial metabolic networks [37]. Despite these advances, a critical gap remains. Most studies are correlative, linking TCM to microbial or metabolic changes without establishing causality. Consequently, the mechanisms by which these changes drive therapeutic effects in distant organs remain unclear. Systematic evidence on whether and how TCM modulates the microbiota–BA–host receptor axis is lacking, a question this review addresses. Elucidating this relationship is essential for understanding TCM and developing microbiome-informed therapies.

The literature search was conducted in PubMed, Web of Science, and Scopus for articles published from January 2010 to December 2024, using combinations of the following keywords: “GM,” “BAs,” “Traditional Chinese Medicine,” “FXR,” “TGR5,” and “gut-X axis.” Studies were included if they examined TCM-mediated modulation of BA profiles or BA receptor signaling in the context of extraintestinal diseases. Both preclinical animal studies and clinical investigations were eligible. Opinion pieces, editorials, and studies not involving TCM were excluded.

This narrative review synthesizes evidence from preclinical models and available clinical studies to propose that microbiota-derived BAs serve as the principal mechanistic bridge through which TCM exerts multi-system protective effects via the gut–X axis. We analyze how specific herbal formulations reprogram BA metabolism and modulate host signaling to ameliorate pathology in cardiovascular, renal, hepatic, skeletal, and endocrine systems. By establishing microbiota–BA–organ axis remodeling as a unifying pathogenic pathway, this review clarifies the molecular basis of TCM and identifies actionable targets for precision interventions in multi-system disorders characterized by gut barrier dysfunction, metabolic dysregulation, and chronic inflammation.

While the majority of studies to date had established correlations between GM alterations, BA profiles, and disease states, establishing definitive causality remained a central challenge. This review evaluated the available evidence, including findings from fecal microbiota transplantation (FMT), antibiotic depletion, and knockout models, which collectively supported a causal role for the microbiota–BA–host receptor axis. We propose this axis as a key, but not exclusive, mechanistic hub. Its centrality stems from its ability to activate a broad network of nuclear and membrane receptors (FXR, TGR5, PXR, VDR) distributed across multiple organ systems, thereby translating microbial ecological changes into coordinated systemic responses.

The gut–X axes discussed in this review (gut–heart, gut–kidney, gut–liver, gut–bone, and gut–endocrine) are defined by the target organ of focus. BA signaling pathways are involved in each of these axes because BAs circulate systemically and their receptors are widely distributed across tissues; the distinct functional outcomes in each axis are determined by tissue-specific receptor expression patterns and the local metabolic context rather than by differences in the BA molecules themselves. The term “gut-endocrine axis” as used here refers to the regulation of glucose homeostasis, insulin sensitivity, and energy expenditure by GM-derived BAs in metabolic disorders such as type 2 diabetes (T2DM) and obesity. These axes serve as an organizational framework for the literature. Throughout the review, the shared central role of BAs as integrative signals and the two fundamental properties of BA signaling (microbiota-dependent plasticity and context-dependent receptor responses) are emphasized to unify these axes conceptually (see Section 3.3).

## 2. BAs as Mechanistic Bridge: How TCM Links GM to Organ Protection

### 2.1. Gut–Heart Axis

#### 2.1.1. GM Remodeling: TCM’s Foundational Cardioprotective Strategy

The GM is now recognized as a fundamental metabolic regulator that governs systemic host physiology and is directly implicated in the pathogenesis of CVDs [38]. This relationship is formally described by the gut–heart axis framework, which encompasses the bidirectional signaling pathways connecting intestinal microbial ecosystems with the cardiovascular system. These pathways critically determine the initiation and advancement of CVDs [38]. GM dysbiosis, characterized by compositional and functional imbalance, acts as a primary instigating factor for the development and progression of major cardiovascular pathologies, including hypertension, atherosclerosis, coronary heart disease, and heart failure (HF) [39]. The mechanistic translation from microbial dysregulation to clinical cardiovascular injury is achieved through three interrelated core processes: disruption of intestinal barrier integrity, induction of systemic immune dysfunction, and establishment of a persistent pro-inflammatory state that directly drives vascular and myocardial damage [40]. Clinical studies in cardiovascular disease patients have identified gut dysbiosis as a pathologically defined state characterized by depletion of beneficial commensal bacteria concurrent with expansion of potentially pathogenic species. This condition can be quantified through specific taxonomic alterations, notably an increased abundance of *Proteobacteria* alongside decreased representation of taxa within the *Firmicutes* phylum, as reported in human cohort studies [41]. In patients with HF, this dysbiotic profile holds clear clinical significance, demonstrating correlation with amplified systemic inflammatory responses and elevated susceptibility to secondary infections [42]. In light of these established pathophysiological connections, therapeutic strategies capable of reestablishing microbial homeostasis present a promising direction for cardiovascular disease (CVD) management.

Within this context, TCM represents a compelling therapeutic paradigm. It functionally modulates gut microbial architecture, promotes the restoration of intestinal homeostasis, and consequently alleviates disease-specific symptoms [43]. Preclinical evidence demonstrates that TCM formulations achieve therapeutic benefits through a spectrum of complementary microbial reprogramming strategies. Baoyuan Decoction (BYD) normalized the *F*/*B* ratio in cardiac hypertrophy [44], while ZeXieYin Formula (ZXYF) improved dyslipidaemia and reduced atherosclerotic plaque by enriching *Akkermansia* and increasing the *F*/*B* ratio [45]. Tianma-Gouteng Granules (TGG) lowered blood pressure through selective modulation of *Desulfovibrio*, *Lachnoclostridium*, and *Turicibacter* [46], and Qing-Xin-Jie-Yu Granule (QXJYG) conferred atheroprotection by enriching *Turicibacter* and *Roseburia* [47]. Collectively, these findings demonstrate that TCM-mediated cardiovascular protection relies on structured ecological reprogramming of the GM.

Importantly, the therapeutic impact of this microbial reprogramming extends beyond community restructuring to influence intestinal barrier integrity. This barrier represents a critical structure whose dysfunction actively drives cardiovascular pathology, creating an essential therapeutic target. Consequently, maintaining GM equilibrium emerges as essential not only for microbial ecology but fundamentally for preserving intestinal barrier function. A healthy GM actively supports barrier integrity through mechanisms including TJ protein maintenance and mucosal immune regulation. In HF pathophysiology, however, compromised cardiac output induces intestinal ischemia and barrier dysfunction, as described in Section 1, facilitating LPS translocation and the resulting chronic low-grade inflammation that accelerates CVD progression [38]. Given this mechanistic cascade, therapeutic restoration of intestinal barrier integrity represents a strategic intervention point along the gut–heart axis [48]. TCM formulations address this target through multifaceted barrier-protective mechanisms. Experimental evidence demonstrates their capacity to enhance intestinal microcirculation, fortify mucosal structure, and decrease epithelial permeability [49]. For example, Naoxintong (NXT) administration in atherosclerotic mice upregulated key TJ proteins, specifically ZO-1, Claudin-1 and Occludin, resulting in reduced LPS translocation and diminished inflammatory cytokine production [50]. Similarly, ZXYF reinforced intestinal barrier integrity through microbiota-dependent mechanisms, improving specific BA profiles including reductions in deoxycholic acid (DCA), thereby attenuating intestinal pathology and systemic inflammation [45].

This barrier restoration occurs in coordination with microbial modulatory effects, representing an integrated two-pronged strategy. While different formulations employ distinct compositional approaches, they converge on a unified therapeutic logic: concurrently enriching beneficial taxa and suppressing pathogenic species to optimize microbial output, while simultaneously upregulating TJ complexes to strengthen physical barrier defense. Notably, the therapeutic impact of gut ecosystem modulation extends beyond these structural changes to encompass functional metabolic outputs. The reconfigured microbial communities subsequently produce modified profiles of bioactive metabolites that directly influence cardiovascular physiology through specific receptor-mediated pathways. These metabolites, which include short-chain fatty acids (SCFAs), trimethylamine N-oxide (TMAO), and BAs, form a crucial additional layer of gut–heart communication. This metabolite-mediated regulation represents a complementary mechanism through which TCM’s reprogramming of the gut ecosystem achieves comprehensive cardiovascular protection.

#### 2.1.2. From TMAO to BAs: TCM Reprograms Cardioactive Gut Metabolites

The therapeutic advantage of TCM in CVD management stems from a dual-pathway strategy that simultaneously targets the intestinal barrier and the metabolic output of gut microbial communities. This approach not only reduces the systemic translocation of harmful microbial products such as LPS by reinforcing barrier function but also actively remodels the microbiota to suppress the endogenous production of detrimental metabolites at their source. This integrated barrier strengthening and metabolite reshaping mechanism embodies a systemic, holistic strategy that addresses multiple facets of CVD pathogenesis concurrently.

**Figure 1:**
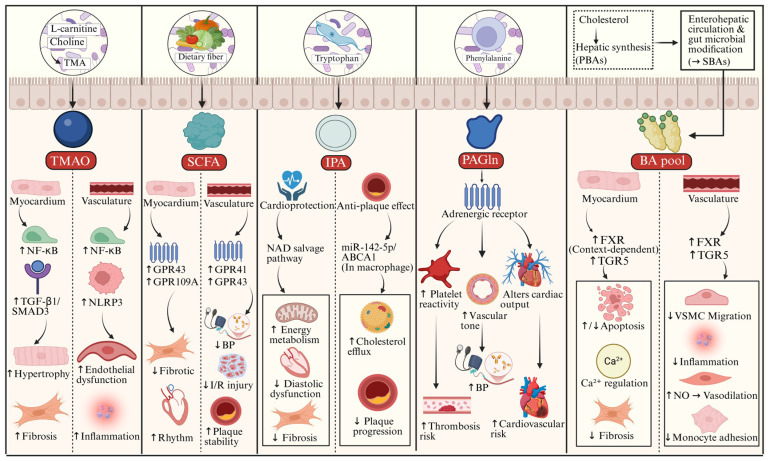
Gut-derived metabolites and cardiovascular signaling. ↑, increase; ↓, decrease. Arrows indicate direction of effect. Created with BioRender (https://biorender.com/).

In this context, the modulation of specific gut-derived metabolites emerges as a critical pathway through which TCM exerts cardiovascular protection. A schematic overview of these metabolite-mediated signaling pathways from the gut to the heart is provided in Figure 1. TMAO has been clinically and mechanistically implicated in CVD, as evidenced by large-scale human cohort studies demonstrating its association with incident cardiovascular events [51]. It promotes myocardial hypertrophy, fibrosis, and atherosclerosis via TGF-β1/SMAD3, NF-κB, and NLRP3 inflammasome activation [4]. TCM interventions lower circulating TMAO: Shenmai Injection and Qing-Xue-Xiao-Zhi Formula alleviated HF and atherosclerosis in part through this mechanism [52,53], and berberine reduced TMAO generation by reshaping gut microbial composition [54]. SCFAs (acetate, propionate, butyrate) provide cardioprotection through GPR41/GPR43/GPR109A signaling, inhibiting myocardial fibrosis, lowering blood pressure, and stabilizing plaques [55,56,57]. Tryptophan-derived IPA enhances cardiac energy metabolism, attenuates atherosclerosis via the miR-142-5p/ABCA1 axis, and mitigates age-related myocardial fibrosis through autophagic restoration [58,59,60,61]. Phenylacetylglutamine (PAGln), a product of bacterial phenylalanine metabolism, modulates cardiovascular risk through adrenergic receptor signaling [62].

While these metabolites represent important axes of gut–heart communication, BAs occupy a key integrative position within this metabolic network, functioning not merely as one among many parallel pathways but as integrative signaling molecules that coordinate multiple regulatory inputs [63]. As introduced in Section 1, BAs act as systemic hormones via receptors including FXR and TGR5, whose roles in cardiovascular physiology are detailed below [64,65]. The complexity of BA signaling is exemplified by FXR’s dual role: its activation can exert both detrimental effects, such as triggering cardiomyocyte apoptosis through mitochondrial dysfunction, and protective influences, including reducing inflammatory enzyme expression and inhibiting vascular smooth muscle cell migration, depending on specific cellular and metabolic contexts [66,67]. Conversely, TGR5 activation consistently promotes cardiovascular protection by enhancing endothelial nitric oxide production, suppressing monocyte adhesion, and regulating cardiomyocyte calcium homeostasis, mechanisms crucial for maintaining vascular function and cardiac contractility [68,69]. Critically, gut microbial BSH activity determines the composition of the BA pool, thereby shaping downstream receptor-mediated signaling [21]. This positions BA metabolism as a pivotal mechanistic hub within the gut–heart axis, where microbial ecology directly interfaces with host cardiovascular physiology [70].

The recognition of BAs as such integrative signals provides a coherent framework to unify TCM’s multifaceted effects. Rather than targeting isolated pathways, TCM functions as a coordinated systems-level regulator that concurrently influences multiple nodes within the host-metabolome network [36]. By strategically recalibrating BA homeostasis through coordinated modulation of microbial biotransformation, hepatic synthesis, and receptor-mediated signaling, TCM formulations such as ZXYF and TGG demonstrated the capacity to orchestrate the complete BA metabolic circuit [45,46]. This systems-level modulation of the BA-centered network thus represents a fundamental mechanism through which TCM translates gut-targeted interventions into systemic cardiovascular benefits, establishing a direct mechanistic bridge between microbial ecology and cardiovascular pathophysiology.

#### 2.1.3. FXR/TGR5 Context-Dependent Modulation: TCM’s Precision Cardioprotection

Emerging evidence identifies BA signaling as a pivotal mechanistic pathway through which TCM achieves cardioprotection via modulation of the gut–heart axis. Representative TCM formulations, including TGG and ZXYF, exemplified this mechanism through orchestrated multi-component interventions that systematically regulated BA homeostasis [45,46]. This regulatory capacity enabled the translation of GM modifications and their metabolic output into measurable cardiovascular benefits, establishing a functional link between intestinal ecology and cardiac physiology.

A core therapeutic strategy common to both TGG and ZXYF involved integrated modulation of the host–microbiota metabolic interactions [45,46]. This shared approach operated through several interrelated mechanisms that worked in concert to restore cardiovascular health. At the microbial level, these formulations restructured gut microbial communities toward a more symbiotic composition, a process exemplified by ZXYF’s significant enrichment of *Akkermansia* (a bacterium known to support intestinal barrier integrity) [45] and TGG’s favorable modulation of genera including *Desulfovibrio* [46]. These microbial modifications simultaneously facilitated the remodeling of BA metabolism, enhancing cholesterol catabolism and increasing fecal BA excretion. Furthermore, both formulations demonstrated potent anti-inflammatory effects by effectively reducing circulating levels of pro-inflammatory cytokines such as IL-1β, IL-6, and TNF-α, while also modulating activity within the renin-angiotensin system [45,46]. Together, these coordinated actions ameliorated endothelial dysfunction and vascular inflammation while optimizing the systemic pool of bioactive BA metabolites.

**Figure 2:**
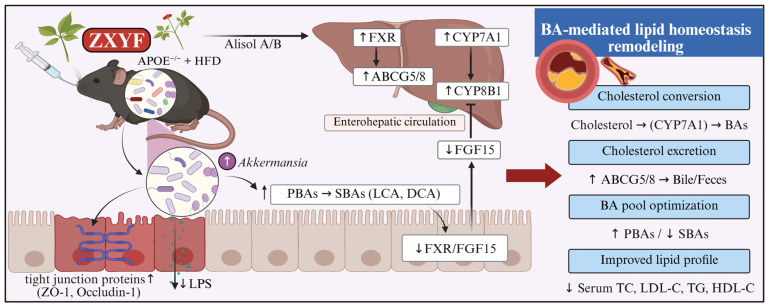
ZeXieYin Formula targets the microbiota-BA-FXR axis to ameliorate atherosclerosis [45]. ↑, increase; ↓, decrease. Arrows indicate direction of effect. Created with BioRender (https://biorender.com/). Abbreviations: BA, bile acid; FXR, farnesoid X receptor.

Despite these foundational similarities, ZXYF and TGG demonstrated distinct, pathology-specific regulatory strategies targeting the FXR signaling pathway, a master regulator of BA synthesis and enterohepatic circulation [45,46]. This strategic divergence reflected a tailored therapeutic approach based on disease context. In experimental models of atherosclerosis, ZXYF employed a dual-tissue modulation strategy to restructure cholesterol and BA metabolism. In the intestinal compartment, ZXYF intervention (via GM remodeling) reduced secondary BAs, thereby downregulating intestinal FXR signaling and decreasing production of the enterokine Fibroblast growth factor 15 (FGF15); this relieved the inhibitory feedback on hepatic BA synthesis, promoting cholesterol conversion to BAs via CYP7A1. Simultaneously in the hepatic compartment, bioactive components within ZXYF (e.g., *Alisol* compounds) acted as direct agonists of hepatic FXR, robustly upregulating the expression of cholesterol efflux transporters ABCG5/8 to enhance direct cholesterol excretion into bile and feces. This coordinated dual-tissue action results in an optimized BA pool, directly addressing the dyslipidemia and metabolic inflammation characteristic of atherosclerosis [45]. The mechanism by which ZXYF targets the microbiota-BA-FXR axis to ameliorate atherosclerosis is illustrated in Figure 2. In contrast, in models of hypertension, TGG adopted a suppressive regulatory mode targeting the FXR pathway [46]. Treatment with TGG downregulated FXR expression in both intestinal and hepatic tissues, leading to reduced FGF15 levels [46]. This suppression subsequently relieved inhibition on CYP7A1 expression, stimulating de novo BA synthesis and altering BA pool distribution. The metabolic and immunomodulatory consequences included significant reductions in key inflammatory mediators such as IL-1β, IL-6, TNF-α, renin, and angiotensin II, culminating in effective blood pressure reduction [46]. The opposing pharmacological effects on the FXR pathway underscored a systems-level therapeutic mechanism inherent to TCM formulations. Rather than applying a uniform intervention, these multi-component prescriptions dynamically recalibrated a central metabolic-inflammatory axis in a context-dependent manner. This precision was facilitated by their complex phytochemical composition, where bioactive compounds interacted with multiple targets within the BA signaling network. The capacity for differential FXR pathway modulation exemplified the potential of TCM for targeted, network-based intervention in complex CVDs.

In summary, ZXYF and TGG illustrate how TCM achieves cardioprotection through shared microbial-metabolic restoration and distinct, pathology-specific FXR modulation strategies [45,46]. These insights reinforce BA signaling as a critical therapeutic target in the gut–heart axis and support the development of microbiota-informed strategies for CVD management.

### 2.2. Gut–Kidney Axis

#### 2.2.1. Dual Targeting of Gut Ecology and Barrier: TCM’s Strategy to Break CKD Cycle

The gut–kidney axis constitutes a complex bidirectional network wherein intestinal microbial communities and renal physiology engage in continuous crosstalk that profoundly influences host metabolic, inflammatory, and immune homeostasis [5]. A substantial body of evidence now implicates intestinal dysbiosis as a critical driver in the pathogenesis of CKD, establishing its relevance across the entire spectrum of disease progression [71]. This pathological association is characterized by a defined sequence of microbial changes. Human studies have consistently shown that patients with CKD consistently display a significant reduction in overall gut microbial diversity (α-diversity), as quantified by lower Chao 1 and Shannon indices, alongside profound compositional shifts within the microbiota [72]. In these patient cohorts, progression towards end-stage renal disease (ESRD) is marked by further taxonomic divergence, with clinical metagenomic analyses reporting up to 190 operational taxonomic units differing from healthy controls, prominently featuring an expansion of families like *Enterobacteriaceae* and *Pseudomonadaceae* [73,74]. A core feature of CKD-associated dysbiosis is the depletion of beneficial genera such as *Bifidobacterium*, *Lactobacillus*, and members of the *Bacteroidaceae* family, concurrent with a bloom of potentially pathogenic *Enterobacteriaceae* species, including *Enterobacter*, *Klebsiella*, and *Escherichia* [75]. Critically, certain bacterial taxa, notably within the *Clostridia* and *Bacilli* classes, possess metabolic pathways for generating uremic toxins. When combined with impaired renal clearance in CKD, these metabolites accumulate systemically, promoting oxidative stress and tubulointerstitial fibrosis, thereby creating a vicious cycle that accelerates kidney injury [76].

Given this central role of the GM, interventions aimed at restoring its ecological balance represent a promising therapeutic frontier. TCM has demonstrated significant potential in this regard through a multi-faceted strategy that includes microbial community restructuring and intestinal barrier restoration. Different TCM formulations reshape the gut ecosystem in pathology-specific ways. Yi-Shen-Hua-Shi granules enhanced microbial diversity and normalized the *F*/*B* ratio [77]; Fufang-Zhenzhu-Tiaozhi prescription (FTZ) corrected dysbiosis by suppressing *Weissella* and *Enterococcus* in DKD [78]; the combination of *Astragalus membranaceus* and *Salvia miltiorrhiza* (AS) enriched SCFA-producing *Akkermansia* and *Lactobacillus* [79]; and QiDiTangShen granules (QDTS) reduced BA-metabolizing bacteria while lowering serum BAs [80].

Beyond compositional restructuring, a crucial and often concurrent mechanism of action involves the restoration of intestinal barrier integrity. Dysbiosis directly undermines the gut’s physical and immune defense, weakening epithelial TJ proteins (e.g., ZO-1, Occludin) and increasing intestinal permeability [5]. This “leaky gut” state facilitates the systemic translocation of bacterial endotoxins and gut-derived uremic toxins, fueling a chronic inflammatory milieu that exacerbates kidney damage [81]. TCM interventions directly counter this barrier dysfunction. The Tangshen formula, for instance, not only reconstructed microbial homeostasis but also reduced serum levels of gut-derived toxins like indoxyl sulfate (IS) and LPS, concomitant with lowering inflammatory markers (MCP-1, TNF-α) in circulation and kidney tissue [82]. The Fufang-zhenzhu-tiaozhi formula (FTZ) strengthened the barrier by upregulating the adhesion protein E-cadherin, and its efficacy being transferable via FMT confirmed the indispensable role of a remodeled microbiota in DKD improvement [78]. Additionally, Shenshuaikang enema provided a direct demonstration of barrier reinforcement by upregulating key TJ proteins (Occludin, Claudin-1, ZO-1), thereby limiting endotoxin leakage and attenuating the associated systemic inflammation to protect renal function [83].

In summary, TCM mediates renal protection via the gut–kidney axis through a coordinated, systems-level approach that simultaneously reconfigures gut microbial ecology and reinforces intestinal barrier function. This dual strategy disrupts the pathogenic cycle of dysbiosis, barrier leak, toxin translocation, and systemic inflammation that perpetuates CKD progression. The therapeutic specificity arises from the ability of different formulations to modulate distinct microbial targets and barrier components based on the underlying renal pathology. Critically, the restoration of a healthy gut ecosystem not only corrects structural and functional deficits but also fundamentally alters the metabolic output of the GM. The modified production of gut-derived metabolites, which serve as direct molecular mediators between the intestine and the kidney, constitutes the next critical layer in understanding how microbial modulation translates into renal outcomes.

#### 2.2.2. Uremic Toxins to BAs: Microbial Metabolites Driving Kidney Injury

The structural and functional remodeling of the GM, as discussed, translates into clinical effects through the production of bioactive metabolites. These molecules serve as direct biochemical messengers in the gut–kidney dialogue, entering systemic circulation to profoundly influence renal pathophysiology. Beyond the well-documented impact of uremic toxins, several key metabolite classes are now recognized for their significant roles, with their effects ranging from detrimental to protective in a context-dependent manner.

TMAO accumulates in CKD and drives renal inflammation and fibrosis via NLRP3 inflammasome activation and macrophage polarization [84,85]. SCFAs counteract these effects by activating GPR43/GPR109A to strengthen the intestinal barrier, reduce endotoxin translocation, and suppress NF-κB-mediated inflammatory signaling [86,87,88,89]. Tryptophan-derived metabolites display a dual role: IPA protects against DKD by preserving mitochondrial function [90], whereas IS and IAA act as protein-bound uremic toxins that can promote inflammation through AhR/p38 MAPK/NF-Κb [91,92,93]. Similarly, tyrosine-derived PCS exacerbates renal oxidative stress and fibrosis via PKC/PI3K pathways [93,94,95,96].

Among these diverse microbial metabolites, BAs emerge as key integrative signaling molecules within the gut–kidney axis. Synthesized from cholesterol and extensively modified by GM, BAs function not merely as digestive surfactants but as systemic hormones [63]. Their biological effects are primarily mediated through nuclear receptors like the FXR and the PXR, as well as the membrane receptor TGR5 [97]. In the kidney, FXR activation exerts multifaceted protection: it modulates lipid and glucose metabolism, suppresses the NF-κB-driven inflammatory cascade, and inhibits fibrotic mediators like TGF-β, thereby mitigating injury in models of acute kidney injury, DN, and CKD progression [98,99,100,101,102]. PXR contributes by improving mitochondrial function [103], regulating uric acid homeostasis [104], and alleviating fibrosis [105]. Concurrently, TGR5 activation promotes mitochondrial biogenesis, reduces oxidative stress, and attenuates renal fibrosis and inflammation in DKD [101].

This regulatory landscape supports the view that BA signaling functions as a core integrative hub within the gut–kidney axis. Unlike metabolites that exert more linear or context-dependent effects, BAs functionally integrate diverse inputs from microbial metabolism through structural modifications and pool composition changes, thereby coordinating a broad receptor-mediated response network. This network directly targets and modulates several core pathological processes driving kidney disease, including metabolic dysregulation, oxidative stress, inflammation, and fibrosis. Consequently, therapeutic modulation of BA metabolism and signaling represents a strategic intervention point that transcends the targeting of individual pathogenic metabolites.

The recognition of BAs as such a central mechanistic node provides a coherent framework to understand and unify the renoprotective effects of TCM. Rather than isolated actions, TCM formulations appear to function as systems-level regulators that strategically rebalance the BA-centered network, bridging upstream microbial ecology with downstream renal pathophysiology. This understanding naturally leads to an examination of how specific TCM formulations operationalize this systems-level perspective through targeted modulation of BA composition and receptor signaling to achieve therapeutic benefits.

#### 2.2.3. Systemic BA Modulation: TCM Paradigm for Diabetic Kidney Disease

The gut–kidney axis has emerged as a critical interorgan communication network that integrates metabolic, inflammatory, and microbial signaling pathways, playing a fundamental role in the pathogenesis of metabolic kidney diseases including diabetic nephropathy (DN). Within this complex regulatory system, BAs function as essential signaling molecules that modulate glucose and lipid homeostasis, regulate inflammatory responses, and influence fibrotic processes through activation of both nuclear and membrane receptors. Dysregulation of BA metabolism represents a key pathological feature in DN, making it a promising therapeutic target. TCM formulations have demonstrated significant potential in modulating BA metabolism through multifaceted pharmacological mechanisms, offering innovative approaches for managing this complex kidney disorder.

A representative example is QiDiTangShen granules (QDTS), which operated through a systemic approach by reshaping GM ecology and normalizing circulating BA profiles. In diabetic *db*/*db* mice, QDTS treatment significantly altered GM composition by reducing BA-metabolizing genera including *Lactobacillus*, *Bacteroides*, and *Roseburia*, while decreasing serum levels of total and secondary BAs [80]. This microbial and metabolic modulation led to amelioration of systemic BA dysregulation and attenuation of renal injury, demonstrating renoprotection through alternative BA receptor pathways and indirect regulation of inflammatory responses.

Future research should focus on establishing clinical translation frameworks that bridge these mechanistic insights to therapeutic applications. Critical directions include validating efficacy in patient subgroups stratified by gut microbial signatures and BA metabolic profiles, developing multi-omics-based network pharmacology maps, and creating pharmacokinetic models that characterize tissue-specific distribution of active compounds. As demonstrated in the QDTS study [80], the formulation’s primary action involved modulating the GM and systemic BA profiles, suggesting that patients with prominent intestinal dysbiosis and elevated serum secondary BAs such as taurocholic acid (TCA), and DCA would derive maximal benefit from such systemic regulatory strategies.

### 2.3. Gut–Liver Axis

#### 2.3.1. GM and Barrier: TCM’s Dual Target for Liver Protection

The gut–liver axis constitutes a vital bidirectional communication network where the liver and gastrointestinal tract interact through vascular, metabolic, and immunological pathways. Central to this axis is the portal venous system, which transports gut-derived microbial products, dietary antigens, and metabolites to the liver for processing. In return, the liver secretes BAs and other regulatory molecules that shape intestinal microbial ecology and mucosal barrier function [6]. This dynamic exchange maintains systemic metabolic and immune homeostasis, and its disruption is a common denominator in the pathogenesis of diverse liver diseases.

Dysfunction of the gut–liver axis manifests primarily through two interconnected pathological processes: gut microbial dysbiosis and impaired intestinal barrier integrity. Altered gut microbial communities, characterized by reduced diversity and shifts in taxonomic composition, directly influence hepatic physiology [106]. For example, specific microbes such as *Bifidobacterium* support immune regulation by promoting regulatory T-cell differentiation, while other microbial components may drive pro-inflammatory Th17 responses [107]. Such microbial imbalances disrupt hepatic metabolism, immune surveillance, and detoxification, contributing to conditions including alcohol-related liver disease (ALD), which is strongly associated with reduced microbial diversity, expansion of pathobionts, and gut virome alterations [108,109].

In this context, TCM offers a systemic therapeutic approach that targets multiple nodes within the gut–liver axis. Rather than acting through a single pathway, TCM formulations often exert hepatoprotective effects by concurrently modulating gut microbial composition and reinforcing intestinal barrier function, thereby interrupting the pathological cascade from gut to liver. Experimental studies illustrate how different TCM formulae corrected dysbiosis in a pathology-specific manner. In models of NAFLD, Ling-Gui-Zhu-Gan decoction (LG) remodeled the GM and enhanced microbial metabolic activity, which correlated with reduced hepatic steatosis [110]. Similarly, Yinzhihuang formula (YZH) enriched beneficial taxa such as *Clostridiales*, *Lachnospiraceae*, and *Bifidobacterium pseudolongum* while suppressing pathogenic genera like *Escherichia-Shigella* and *Serratia*. These effects have been confirmed as microbiota-dependent through FMT [111]. Other formulae, including Sanwei Ganjiang Powder (SWGJ) and Danggui Shaoyao San (DSS), restored microbial balance by increasing *Lactobacillus* and *Bifidobacterium* or enhancing beneficial *Bacteroidota*, respectively, thereby mitigating liver injury through the gut–liver axis [112,113].

Critically, gut dysbiosis alone is often insufficient to induce significant hepatic damage; it typically requires the concomitant breakdown of intestinal barrier integrity. The intestinal barrier is not a passive structure but an active immunomodulatory site. When compromised due to dysbiosis or other insults, it permits translocation of bacterial endotoxins such as LPS and metabolites into the portal circulation, directly activating hepatic immune cells and triggering persistent inflammation [114]. This barrier dysfunction thus acts as an essential amplifier linking gut ecology to liver pathology.

Several TCM formulations directly addressed this barrier component. Gegen-Qinlian Decoction (GQD) not only increased *Akkermansia* and reduced *Desulfovibrio* but also repaired TJ proteins, thereby reducing endotoxemia and improving BA metabolism [115]. LG upregulated intestinal TJ protein expression, limiting LPS translocation and subsequent hepatic inflammation [110]. YZH preserved epithelial structure and mucus integrity, supporting bile flow and protection against cholestatic injury [111], while SWGJ restored ZO-1 expression to maintain barrier architecture [112]. The molecular link between barrier disruption and hepatic inflammation often involves pattern-recognition receptors such as TLR4. Upon recognizing translocated LPS, TLR4 activates downstream NF-κB and MAPK signaling, driving the production of pro-inflammatory cytokines that fuel liver injury [116]. In conditions like sepsis or chronic alcohol exposure, barrier dysfunction is further exacerbated by downregulation of TJ proteins and TLR4-mediated epithelial apoptosis, creating a vicious cycle of endotoxemia, systemic inflammation, and metabolic dysregulation [6,117]. TCM interventions break this cycle by simultaneously strengthening the physical barrier and modulating inflammatory signaling, as evidenced by the ability of formulae like LG to attenuate oxidative stress and inflammatory cytokine expression in the liver [110].

Thus, TCM achieves hepatoprotection through coordinated modulation of gut microbial ecology and intestinal barrier function. This systems-level intervention restructures microbial communities and reinforces barrier integrity, thereby reshaping gut-derived metabolite profiles. These metabolites, including BAs, SCFAs, and tryptophan derivatives, serve as key molecular mediators linking gut modifications to hepatic benefits.

#### 2.3.2. SCFAs, TMAO, BAs: Microbial Metabolites Driving Liver Disease

The structural and functional remodeling of the GM, as previously discussed, translates into clinical effects through the production of diverse bioactive metabolites. These molecules serve as direct biochemical messengers in the gut–liver dialogue, entering portal circulation to profoundly influence hepatic pathophysiology. Several key metabolite classes have been identified with significant, though distinct, roles in liver disease progression.

SCFAs enhance hepatic health by upregulating TJ proteins to reinforce the gut barrier and by suppressing hepatic lipogenesis and inflammation via LKB1-AMPK-Insig and NF-κB pathways [118,119,120,121]. TCM formulas such as LG and DSS elevate fecal SCFAs, contributing to their hepatoprotective effects [110,113]. In contrast, TMAO promotes NAFLD by driving hepatic lipid accumulation, sinusoidal endothelial dysfunction, and pro-inflammatory macrophage polarization, with its production amplified by intestinal IL-33 in a feed-forward loop that accelerates MASLD progression [122,123]. Tryptophan-derived indole derivatives activate AhR to strengthen the intestinal barrier and inhibit hepatic stellate cell activation, illustrating a protective role in limiting fibrogenesis [124].

Among these diverse microbial metabolites, BAs occupy a central integrative position within the gut–liver metabolic network [125]. Building on the BA receptor framework established in Section 1, FXR activation in the liver exerts hepatoprotective effects by suppressing BA synthesis via CYP7A1 feedback inhibition and repressing inflammatory and fibrotic signaling [25,126]. PXR contributes to hepatic homeostasis by modulating immune responses, including suppression of pro-inflammatory M1 macrophage polarization [127]. VDR activation inhibits hepatic stellate cell activity, with its deficiency linked to increased inflammation and fibrosis [128]. Additionally, TGR5 signaling modulates immune and inflammatory processes, including inhibition of NLRP3 inflammasome activation [129]. Through this multi-receptor network, BAs orchestrate coordinated regulation of hepatic metabolism, inflammation, and fibrogenesis.

This regulatory network established BA signaling as an integrative microbiota-dependent hub within the gut–liver axis. BAs integrated microbial inputs into a multi-receptor network directly targeting metabolic dysregulation, inflammation, and fibrosis as core drivers of liver disease. Therapeutic modulation of BA metabolism thus represented a strategic intervention point. This framework explained TCM hepatoprotection, wherein formulations rebalance the BA-centric network through coordinated adjustments to BA pool composition, receptor expression, and downstream signaling, revealing their therapeutic potential for complex liver disorders. At the same time, sustained supraphysiological FXR activation can paradoxically worsen cholestatic injury or promote hepatocyte apoptosis [130], and the accumulation of cytotoxic secondary BAs such as DCA is a recognized risk factor for carcinogenesis [131], which highlights the necessity of calibrated rather than maximal modulation of this axis.

#### 2.3.3. Three TCM Patterns of BA Modulation in MASLD, Cholestasis, and Fibrosis

Gut–liver Axis dysregulation underlies MASLD, cholestasis, and fibrosis. TCM formulations target this axis by modulating microbiota, reinforcing intestinal barriers, and reprogramming BA metabolism with cytoprotective effects [106]. Analysis of representative TCM formulations reveals three distinct yet complementary mechanistic patterns for targeting the gut–liver axis.

##### Metabolic Axis Regulation Through GM-BA Synchronization

**Figure 3:**
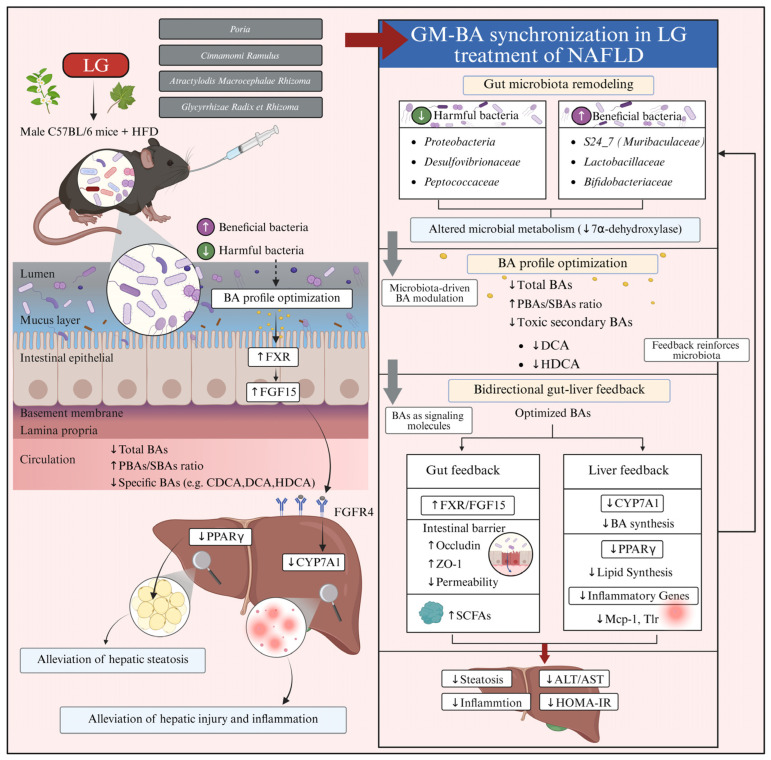
Ling-Gui-Zhu-Gan Decoction ameliorates NAFLD through synchronized modulation of the GM-BA axis [110]. LG was orally administered to HFD-fed male C57BL/6 mice. In the gut lumen, LG remodels the GM by reducing harmful bacteria and enriching beneficial taxa. This increases the primary-to-secondary BA ratio and reduces cytotoxic secondary BAs (↓ DCA, ↓ HDCA). Optimized BAs activate ileal FXR (↑ FXR) and FGF15 (↑ FGF15), which enter the portal circulation and downregulate hepatic CYP7A1 (↓ CYP7A1) and lipogenic genes (↓ PPARγ), thereby ameliorating hepatic steatosis, inflammation, and insulin resistance. ↑, increase; ↓, decrease. Arrows indicate direction of effect. Created with BioRender (https://biorender.com/). Abbreviations: CYP7A1, cholesterol 7α-hydroxylase; DCA, deoxycholic acid; FGF15, fibroblast growth factor 15; FXR, farnesoid X receptor; HDCA, hyodeoxycholic acid; HFD, high-fat diet; LG, Ling-Gui-Zhu-Gan Decoction; GM, gut microbiota; BA, bile acid; NAFLD, nonalcoholic fatty liver disease; PPARγ, peroxisome proliferator-activated receptor gamma.

LG demonstrated this pattern in MASLD by reducing serum total BAs and secondary BAs such as DCA and hyodeoxycholic acid (HDCA), normalizing the primary-to-secondary BA ratio while enhancing intestinal barrier integrity and suppressing hepatic lipogenesis via PPARγ downregulation [110]. The formulation further activated ileal FGF15/FXR signaling while inhibiting hepatic CYP7A1 expression [110]. The schematic overview of this gut–liver bidirectional regulatory mechanism is illustrated in Figure 3. YZH complemented this approach in cholestatic conditions by reinforcing intestinal barrier through TJ upregulation and promoting BA excretion. Downregulation of CYP7A1, CYP7B1, and CYP27A1 reduced hepatotoxic BAs, including DCA, LCA, TCA, and T-β-MCA [111].

##### Integrated Protection Through Antioxidant and Anti-Inflammatory Pathway Activation

This pattern was demonstrated by SWGJ, which restored GM balance while enhancing intestinal barrier function in models of drug-induced dysbiosis and chronic liver injury [112]. SWGJ upregulated critical BA transporters including NTCP, BSEP, and MRP2, while activating the Nrf2 antioxidant pathway via Bach1 inhibition [112].

##### Specialized BA Regulation for Advanced Hepatic Disorders

Da-Chai-Hu Decoction (DCHD) exemplified this approach by reducing the enlarged BA pool and decreasing pro-inflammatory conjugated BAs through coordinated upregulation of hepatic FXR and BSEP expression, coupled with downregulation of CYP7A1 and NTCP and a strategic shift toward alternative BA synthesis pathways [132]. Complementing this, Bao-Gan-Ning Decoction (BGN) promoted efficient BA circulation through synergistic regulation of hepatic and intestinal transporters while activating the PPARα/CYP7A1 pathway [133]. In metabolic steatohepatitis, Ge-Gen-Qin-Lian Decoction (GQD) implemented a comprehensive strategy by simultaneously correcting dysbiosis, fortifying the intestinal barrier, and reprogramming BA metabolism, with therapeutic efficacy correlating with restoration of colonic TGR5 and VDR expression and enrichment of BA derivatives that function as receptor ligands [115].

These diverse formulations collectively demonstrate a systems-level therapeutic approach through convergent core strategies while exhibiting specialized pathway engagements that define their clinical indications. The fundamental axis-targeting mechanisms shared across formulations include enrichment of BA-metabolizing probiotic bacteria, upregulation of TJ proteins to reinforce intestinal barrier integrity, precise modulation of BA synthesis and transport pathways, and integration of anti-inflammatory and antioxidant signaling systems [110,111,112,113,115,132,133,134]. The strategic specialization of each formulation emerges from differential emphasis on specific molecular pathways: LG primarily targeted hepatic metabolic dysregulation through PPARγ modulation combined with SCFA-mediated gut microenvironment improvement [110]; YZH was specialized for cholestatic conditions, focusing on barrier reinforcement and enhanced BA excretion pathways [111]; SWGJ uniquely integrated Nrf2-mediated antioxidant defenses with coordinated transporter regulation [112]; DCHD demonstrated particular efficacy in FXR-mediated BA pool normalization and detoxification [132]; BGN emphasized PPARα-CYP7A1 metabolic axis activation to optimize BA circulation [133]; and GQD achieved comprehensive microbiota-receptor axis reprogramming specifically tailored for metabolic liver disease [115]. These findings inform novel hepatology therapeutics. Future research should prioritize bioactive compound identification, clinical validation, and development of BA-centric, microbiome-informed approaches for personalized liver disease management.

### 2.4. Gut–Bone Axis

#### 2.4.1. Leaky Gut to Inflamed Joint: TCM Restores Barrier to Treat Arthritis

Emerging research has established a critical connection between GM dysbiosis and the pathogenesis of various bone-related disorders, including osteoarthritis (OA), rheumatoid arthritis (RA), and osteoporosis (OP). This relationship is characterized by distinct, disease-specific microbial alterations. For instance, studies in the elderly population have identified increased abundances of *Actinomyces*, *Eggerthella*, and *Lactobacillus*, alongside reductions in *Escherichia*, *Shigella*, and *Veillonella* in OP contexts [135]. In RA, a notable microbial signature is the early and sustained expansion of *Prevotella* species, particularly *Prevotella copri*, which correlates with disease severity [136]. These compositional shifts are not merely associative; dysbiosis actively contributes to disease by modulating host immune activity, sustaining chronic inflammation, and disrupting bone metabolic homeostasis [137].

The path from gut dysbiosis to joint pathology involves several sequential and interconnected mechanisms. The initial and pivotal step is the disruption of intestinal barrier integrity. Dysbiosis can impair the expression and function of TJ proteins, increasing intestinal permeability. This breakdown allows for the translocation of microbial components into systemic circulation. A key mediator in this process is Zonulin, a regulator of TJs whose inhibition has been shown to reduce arthritis severity in preclinical models [137,138]. The systemic dissemination of gut-derived products, particularly bacterial endotoxins like LPS, constitutes the next critical link. Once in circulation, LPS can activate pattern recognition receptors such as TLRs on immune and joint cells. This sustained activation triggers downstream signaling cascades, including the NF-κB pathway, leading to the production of pro-inflammatory cytokines, such as TNF-α, IL-1β, and IL-6. In the joint, this inflammatory milieu promotes synovial hyperplasia, cartilage matrix degradation, and bone destruction, driving the progression of both RA and OA [139,140].

Within this mechanistic framework, TCM demonstrates therapeutic potential by targeting multiple nodes along the gut–bone axis. Experimental evidence indicates that TCM interventions can concurrently restore intestinal barrier function and modulate downstream inflammatory signaling. For instance, Jingfang Granule (JFG) not only suppressed the NLRP3 inflammasome and TLR4/NF-κB signaling to reduce systemic and joint inflammation in RA models but also directly upregulated intestinal TJ proteins such as Claudin-5 and ZO-1, thereby addressing a root cause of endotoxin translocation [141]. Similarly, Licorice (GC) has been reported to restore barrier function in collagen-induced arthritis models by enhancing TJ protein expression and regulating microbial metabolites, thereby helping to rebalance the gut–bone–immune axis [142]. Furthermore, *Angelica sinensis* polysaccharide (ASP) reinforced the intestinal barrier by enhancing Claudin-5 expression and, through the upregulation of factors like Slit3 and Rgs18, directly suppressed osteoclast differentiation to promote bone formation, showcasing a dual barrier-protective and bone-anabolic effect [143].

TCM protects against bone disorders by fortifying the intestinal barrier and modulating joint inflammation, underscoring gut homeostasis as central to musculoskeletal health. These effects are mediated through gut barrier restoration and microbial remodeling, which reshape GM-derived metabolites. SCFAs, tryptophan derivatives, and BAs regulate bone cell activity, inflammation, and immune responses. Elucidating this metabolite-host interplay provides a molecular basis for TCM’s therapeutic effects on the gut–bone axis.

#### 2.4.2. TMAO, SCFAs, BAs: Microbial Metabolites Shaping Bone Health

GM-derived metabolites serve as critical biochemical messengers, translating microbial ecological changes into direct effects on bone and joint homeostasis. Among these, several key classes exert significant but distinct influences on skeletal health, with their regulatory roles ranging from detrimental to protective.

TMAO promotes vascular calcification and joint inflammation by activating NLRP3/NF-κB and polarizing macrophages toward a pro-inflammatory phenotype [135,144,145], whereas SCFAs maintain Treg/Th17 balance, inhibit osteoclastogenesis, and protect cartilage by suppressing NF-κB and MAPK pathways [146,147]. In RA models, TCM formulas such as JFG elevated SCFA levels and attenuated inflammation via AMPK-mediated suppression of ferroptosis [141], while Zhubi Decoction exerted similar immunomodulatory effects [148]. Other metabolites exhibit context-dependent roles: gut-derived serotonin inhibits osteoblast proliferation, whereas CNS serotonin promotes bone formation [149,150]; polyamines support bone strength by promoting osteoblast activity and driving M2 macrophage polarization [151,152,153].

Among these diverse signaling molecules, BAs have emerged as critical integrative regulators within the gut–bone axis. Beyond their canonical metabolic functions, BAs exert multifaceted effects on bone homeostasis through systemic receptor signaling. For instance, secondary BAs help orchestrate the critical balance between Treg and Th17 cells, an immunological equilibrium essential for maintaining bone health [135]. In OA, reduced abundance of specific bacteria such as *Clostridium bolteae* leads to decreased production of ursodeoxycholic acid (UDCA), a BA precursor that normally protects bone by antagonizing intestinal FXR signaling [7]. Moreover, BAs like DCA and LCA can suppress TNF-α production from macrophages via FXR-dependent mechanisms, thereby contributing to the control of inflammation [154]. Beyond FXR, activation of the membrane receptor TGR5 by BAs such as HDCA induces anti-inflammatory M2 polarization in bone marrow-derived macrophages, inhibits NF-κB signaling, and has been shown to mitigate estrogen-associated bone loss while promoting osteoblast differentiation [155,156]. BAs also facilitate the intestinal absorption of vitamin D, a critical regulator of calcium homeostasis and bone mineralization [157,158]. Notably, certain secondary BAs may act as ligands for the VDR, potentially exerting direct regulatory effects on bone metabolism [150].

BA signaling integrates metabolic, inflammatory, and immune inputs through a microbiota-dependent receptor network targeting the core pathological triad of bone disorders. Therapeutic modulation of BA homeostasis thus transcends targeting individual metabolites. This framework positions BAs as a central node for understanding TCM osteoprotection, wherein formulations recalibrate this network to bridge microbial ecology with skeletal pathophysiology.

#### 2.4.3. THDCA-TGR5: A BA-Mediated Strategy for RA

The gut–bone–immune axis plays a central role in RA pathogenesis. Recent research demonstrates how Fuzi (*Aconitum carmichaelii* lateral root), a traditional Chinese herb with recognized warming properties, modulates this axis through BA metabolism to treat cold-exacerbated RA. This work establishes Fuzi as a microbiota-targeted therapeutic with defined mechanisms of action [159].

##### Selective Modification of GM and BA Profile

Fuzi administration corrected cold-induced dysbiosis by increasing the abundance of *Lachnospiraceae* and *Ruminococcaceae* families. These bacteria are key producers of secondary BAs. Targeted metabolomics confirmed that Fuzi treatment elevated systemic levels of the secondary BA taurohyodeoxycholic acid (THDCA), while primary BAs remained unchanged [159].

##### THDCA-Activated Anti-Inflammatory Pathway in Joint Tissues

THDCA bound and activated the TGR5 receptor on immune cells. This activation increased intracellular cAMP levels and PKA activity, which subsequently inhibited NLRP3 inflammasome formation in synovial tissues. The resulting reduction in IL-1β and IL-18 production contributed to decreased joint inflammation and bone erosion.

##### Microbiota-Dependent Mechanism Confirmed by Transplantation

FMT experiments provided causal evidence: transferring gut bacteria from Fuzi-treated donors to arthritic recipients normalized the recipients’ gut dysbiosis and replicated both the metabolic changes and therapeutic benefits. Recipients showed elevated THDCA levels, reduced arthritis scores, and preserved bone structure [159].

The findings support developing microbiota-focused therapies, including specific probiotic formulations or THDCA-based supplements, for immune-mediated bone disorders. By connecting traditional use with modern mechanistic understanding, this work advances integrative approaches to RA management.

### 2.5. Gut–Endocrine Axis

#### 2.5.1. Gut Ecology and Barrier: TCM’s Dual Strategy for Diabetes and Obesity

The GM plays a central role in regulating host metabolism, with dysbiosis increasingly recognized as a critical factor in the development of metabolic disorders [12]. Within this context, the term “gut-endocrine axis” as used in this review refers to the GM-BA-mediated regulation of glucose homeostasis, insulin sensitivity, and energy expenditure, with a focus on obesity and T2DM. In T2DM patients, this metabolic disease-associated dysbiosis manifests through characteristic compositional shifts documented in multiple cohort studies. Specifically, affected individuals exhibit an elevated proportion of facultative anaerobic bacteria alongside a consistent reduction in beneficial butyrate-producing genera including *Faecalibacterium*, *Clostridium*, and *Akkermansia* [160]. These microbial alterations are not merely correlative markers of disease; functional studies demonstrate they actively contribute to metabolic dysfunction. The causal relationship is supported by interventional studies using specific bacterial strains. For example, administration of *Akkermansia muciniphila* has been shown to improve obesity-related parameters in overweight and obese human volunteers, providing direct clinical evidence that restoring beneficial microbial populations can ameliorate metabolic disturbances and highlighting the therapeutic potential of targeting the GM [161].

Building upon this understanding, TCM has emerged as a promising approach for metabolic disorders, with multiple formulations reshaping the gut ecosystem and correlating with metabolic improvement. Jiang-Tang-San-Huang pill (JTSH) enhanced insulin sensitivity through modulation of *Bacteroides*, *Bifidobacterium*, and *Lactobacillus* [162]; Shouhui Tongbian (SHTB) ameliorated glucose tolerance and insulin resistance by enriching *Parabacteroides* and *Akkermansia* [163]; Simiao Wan (SMW) improved insulin resistance and hepatic steatosis while enriching *Allobaculum*, *Akkermansia*, and *Lactobacillus* [164]; PuRenDan (PRD) reversed T2DM-associated dysbiosis with a restored *F*/*B* ratio [165]; Zhi-Kang-Yin (ZKY) promoted *Akkermansia* proliferation with corresponding glycemic improvement [166]; and Kang Shuai Lao Pian (KSLP) corrected obesity-induced dysbiosis by restoring microbial diversity and suppressing *Proteobacteria* [167].

Beyond compositional changes in microbial communities, the intestinal microbiota further contributes to metabolic dysregulation through its impact on intestinal barrier integrity [168]. Studies in obese and diabetic animal models have reported impaired barrier function, often characterized by reduced expression of TJ proteins and elevated circulating endotoxin levels [169]. This “leaky gut” state, which can be induced by microbiota from obese hosts, may allow translocation of bacterial LPS into systemic circulation. The resulting endotoxemia has been associated with low-grade systemic inflammation and altered glucose regulation, potentially contributing to a cycle that exacerbates metabolic disturbances [170]. Within this pathological framework, TCM offers therapeutic interventions that target barrier dysfunction through distinct but complementary mechanisms. Direct reinforcement of TJ complexes represents one primary approach. GQD significantly increased expression of the TJ protein Occludin in the colon, thereby reinforcing intestinal barrier integrity, suppressing inflammation, and alleviating insulin resistance [171]. Building upon barrier reinforcement, some formulations additionally target the upstream drivers of barrier disruption. Xiehuo-Guzheng (XHGZ) granules regulated GM composition, relieved ileum histopathological damage, and increased expression of multiple TJ proteins including Occludin, ZO-1, and Claudin-1, while concurrently reducing circulating LPS content. These coordinated actions suggest that the anti-diabetic, anti-β cell dedifferentiation, and anti-inflammatory effects of XHGZ granules were mediated through integrated modulation of GM and their metabolic products [172]. Extending these concepts to prevention of microbial translocation, Shen-Yan-Fang-Shuai formula (SYFSF) effectively prevented translocation of intestinal bacterial products into the bloodstream by restoring intestinal barrier integrity and reducing permeability, thereby alleviating the systemic inflammation and insulin resistance triggered by this pathological process [173].

Taken together, these lines of evidence suggest that the GM influences host metabolic homeostasis through two interconnected pathways: compositional remodeling that alters microbial metabolic output, and functional changes in the intestinal barrier that may permit systemic translocation of pro-inflammatory microbial products. This dual-pathway understanding provides a mechanistic rationale for TCM strategies that target multiple nodes along the gut–endocrine axis. Reshaping GM and barrier function alters host metabolism by modulating GM-derived metabolites including SCFAs, BCAAs, TMAO, tryptophan derivatives, and BAs. These molecules mediate communication between the gut ecosystem and systemic metabolism, exerting protective or pathogenic effects on glucose homeostasis and insulin sensitivity. Elucidating their roles in metabolic regulation is critical for understanding the gut–endocrine axis and provides a molecular basis for TCM’s therapeutic effects.

#### 2.5.2. SCFAs, BCAAs, BAs: Microbial Metabolites Linking Gut to Metabolism

The intestinal microbiota influences host metabolic homeostasis through the production of bioactive metabolites that function as signaling molecules between the gut microbial ecosystem and host metabolic pathways. These metabolites demonstrate distinct regulatory effects ranging from protective to pathogenic influences on glucose metabolism and insulin sensitivity.

SCFAs enhance insulin sensitivity by stimulating incretin secretion and reinforcing the intestinal barrier [174,175]; TCM interventions such as *Morus alba* extracts and XHGZ enrich SCFA-producing bacteria and elevate circulating SCFAs [172,176]. Excessive BCAAs, modulated by gut microbial metabolism, are associated with insulin resistance, and dietary isoleucine restriction rapidly restores metabolic homeostasis in obese models [177,178]. TMAO impairs pancreatic β-cell function through mitochondrial impairment and inflammatory pathway activation, contributing to T2DM pathogenesis [179]. Tryptophan catabolism is partitioned by the GM into the kynurenine pathway (correlated with insulin resistance) or indole derivative production (protective), suggesting that modulating this balance could offer a novel metabolic regulation strategy [180,181,182].

In the context of metabolic regulation, BAs act as endocrine signaling molecules that link GM to host metabolism [183]. In disorders such as T2DM and obesity, alterations in gut microbial composition lead to BA pool modifications characterized by reduced secondary BA formation, diminishing BA receptor activation and impairing critical metabolic functions, thereby establishing a direct connection between microbial ecology and endocrine signaling [184]. For instance, intestinal FXR activation induces FGF19/FGF15 expression, improving insulin sensitivity and glucose tolerance, the disorder of intestinal flora leads to a decrease in the generation of secondary BAs, and there with the activation of BA receptors cut down, which further leads to the disorder of glucose metabolism and the occurrence of T2DM [185]. while TGR5 activation, particularly by DCA and LCA, stimulates GLP-1 secretion from intestinal L cells to enhance postprandial glycemic control [186]. Thus, dysregulation of the BA-FXR/TGR5 signaling axis represents a fundamental mechanistic link between gut dysbiosis and metabolic disease progression. This framework establishes BA signaling as an integrative component coordinating glucose metabolism and energy homeostasis through core endocrine pathways, providing a conceptual basis for understanding TCM wherein formulations recalibrate BA-centered networks. Specific TCM interventions modify GM and BA profiles to activate FXR and TGR5 signaling, translating ecological modifications into metabolic improvements.

#### 2.5.3. JTSH vs. SMS: Divergent BA Profiles Converge on FXR/TGR5 for Metabolic Benefits

The GM-BA axis has been established as a fundamental regulator of host metabolism through recent research. This understanding provides a valuable framework for elucidating the therapeutic mechanisms of traditional herbal medicine in metabolic disorders. Evidence demonstrates that classical herbal formulae, including JTSH pill and Shengmai San (SMS), achieved their metabolic benefits through modulation of this axis [162,187]. A comparative analysis reveals that these formulae employed common foundational strategies while executing distinct mechanistic programs, leading to specialized therapeutic outcomes.

All examined herbal interventions shared a core therapeutic approach involving functional modification of the gut ecosystem. Each formula induced consistent and significant alterations in gut microbial composition, which subsequently drove specific remodeling of host BA metabolism. JTSH treatment enriched bacterial genera with BSH activity, including *Bacteroides* and *Lactobacillus* [162]. Conversely, SMS administration selectively reduced the abundance of BSH-active *Lactobacillus* [187]. These microbial modifications represented a shared initial step in the therapeutic action of these formulae. The altered microbial activity led to formula-specific changes in the BA pool. The microbial shift promoted by JTSH resulted in ileal accumulation of unconjugated BAs, particularly chenodeoxycholic acid (CDCA) and DCA [162]. In contrast, the microbial changes induced by SMS led to a systemic increase in tauro-conjugated BAs, with TCA showing the most pronounced elevation [187]. Despite these divergent effects on BA profiles, both interventions demonstrated capacity for targeted metabolic modulation. Both JTSH and SMS ultimately engaged key BA-sensing receptors, primarily the FXR and TGR5 [162,187]. This convergence at the receptor level represented a shared functional endpoint, effectively linking microbial ecology to host metabolic physiology. The engagement of these receptors provided a common mechanistic pathway through which microbial modifications translated into physiological effects.

**Figure 4:**
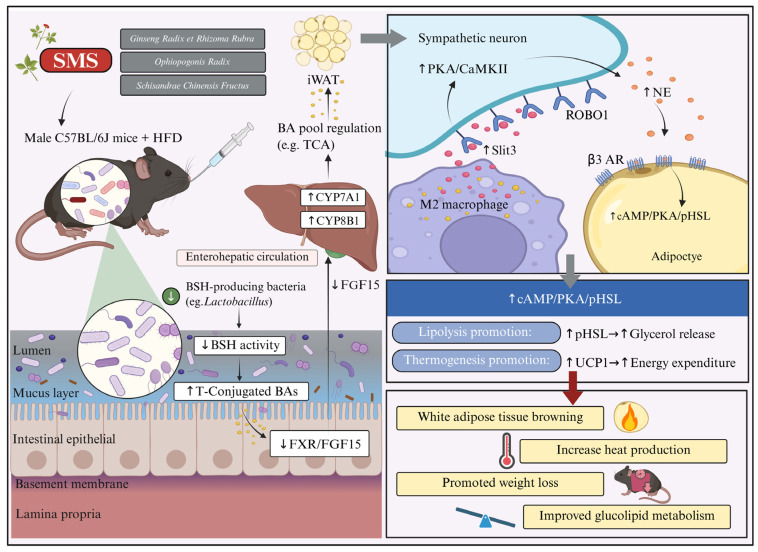
Shengmai San alleviates obesity through GM-BA-macrophage-sympathetic axis [187]. SMS was orally administered to HFD-fed male C57BL/6 mice. SMS reduces BSH-active bacteria (↓ *Lactobacillus*), decreasing ileal BSH activity and increasing tauro-conjugated BAs (↑ TCA). Elevated TCA inhibits ileal FXR-FGF15 signaling (↓ FXR, ↓ FGF15), relieving repression of hepatic BA synthesis (↑ CYP7A1). In inguinal white adipose tissue (iWAT), TCA promotes M2 macrophage polarization. M2 macrophages secrete Slit3, which activates sympathetic neurons via ROBO1 (↑ PKA/CaMKII), enhancing norepinephrine release (↑ NE). NE drives lipolysis (↑ pHSL) and thermogenesis (↑ UCP1), promoting white adipose tissue browning and reducing body weight. ↑, increase; ↓, decrease. Arrows indicate direction of effect. Created with BioRender (https://biorender.com/). Abbreviations: BSH, bile salt hydrolase; CaMKII, calcium/calmodulin-dependent protein kinase II; CYP7A1, cholesterol 7α-hydroxylase; FXR, farnesoid X receptor; FGF15, fibroblast growth factor 15; HFD, high-fat diet; iWAT, inguinal white adipose tissue; NE, norepinephrine; pHSL, phospho-hormone-sensitive lipase; PKA, protein kinase A; ROBO1, roundabout receptor 1; SMS, Shengmai San; TCA, taurocholic acid; UCP1, uncoupling protein 1. The Latin names of the three herbs in this figure follow the pharmacopoeial standard for medicinal materials, which differs from the botanical nomenclature.

JTSH functioned as a systemic metabolic coordinator through its unique mechanism. The elevated unconjugated BAs served as potent dual agonists, simultaneously activating intestinal FXR and TGR5. FXR activation induced FGF15 expression, which suppressed hepatic BA synthesis through downregulation of CYP7A1 and CYP8B1. Concurrent TGR5 activation stimulated glucagon-like peptide-1 (GLP-1) secretion from enteroendocrine L cells. This coordinated dual-pathway activation orchestrated comprehensive improvements in glucose homeostasis, insulin sensitivity, and systemic inflammation, making JTSH particularly effective for the complex pathology of T2DM [162]. SMS operated through a distinctive adipose tissue-centric mechanism. The formula-induced elevation of TCA functioned as a critical signaling mediator within inguinal white adipose tissue (iWAT). TCA promoted polarization of resident macrophages toward an anti-inflammatory M2 phenotype. These M2 macrophages subsequently secreted Slit3, which activated the protein kinase A/calcium/calmodulin-dependent protein kinase II (PKA/CaMKII) signaling pathway in local sympathetic neurons through binding to the Roundabout receptor 1 (ROBO1). This neuro-immune communication enhanced norepinephrine release, stimulating lipolysis and inducing uncoupling protein 1 (UCP1) expression in adipocytes, thereby driving white adipose tissue browning and thermogenesis [187]. The complete mechanistic cascade of SMS-mediated obesity alleviation via the GM-BA-macrophage-sympathetic axis is illustrated in Figure 4. Additional herbal formulae demonstrated further mechanistic diversity within this regulatory framework. GQD primarily restored gut microbial balance and normalized BA homeostasis, leading to activation of the TGR5-cAMP-PKA-CREB pathway and consequent enhancement of GLP-1 secretion for glycemic control [188]. Red Ginseng Extract (RGS) exerted its insulin-sensitizing and anti-obesity effects mainly through direct activation of ileal TGR5, promoting GLP-1 release and stimulating systemic energy expenditure [189].

Herbal formulae targeting the GM-BA axis function as ecological modulators, reshaping microbial communities to generate context-specific BA profiles that regulate receptor-mediated programs in target tissues. Future priorities include human validation via clinical trials with multi-omics profiling, causality establishment using germ-free models and tissue-specific knockouts, and intervention personalization based on individual microbial and metabolic phenotypes. Understanding shared and unique therapeutic signatures will advance precision herbal medicine for metabolic disorders.

### 2.6. Liver as Metabolic Processor: Integrating Gut Signals in TCM Pharmacology

Current evidence demonstrates the liver’s essential role as a central metabolic processor within gut–organ communication networks. The portal venous system creates a direct anatomical pathway through which gut-derived metabolites, microbial components, and BA precursors first reach the liver for processing before systemic distribution. This functional relationship positions the liver as a critical regulator that modulates, integrates, and amplifies intestinal signals. The liver executes this integrative function through three primary mechanisms: the synthesis of primary BAs via CYP7A1 under FXR regulation; the biotransformation of gut-derived metabolites such as trimethylamine to TMAO; and the immune-mediated clearance of gut-translocated bacterial components by Kupffer cells. These biochemical processes collectively determine the systemic bioavailability and signaling characteristics of intestinal outputs.

The therapeutic significance of hepatic integration is evident across various disease contexts. In cardiovascular pathology, the protective effects mediated through the gut–heart axis involve hepatic processing. The ZXYF achieved cardioprotection not only through GM remodeling but also via coordinated modulation of hepatic CYP7A1 expression and BA signaling [45]. Similarly, in renal disease, hepatic detoxification of intestinally derived uremic toxins represents a crucial protective mechanism. QiDiTangShen Granules exerted renoprotective effects partly through hepatic-mediated normalization of systemic BA profiles [80]. In metabolic disorders, the JTSH modulated GM to reshape BA profiles, which subsequently activated hepatic FXR and systemic TGR5 signaling to improve insulin sensitivity [162]. These findings illustrate how different TCM formulations employed a common strategy of leveraging the liver’s position to translate gut ecological modifications into targeted physiological responses in distant organs.

Thus, the liver serves as the central hub in gut–organ communication, converting intestinal signals into systemic outputs. This framework explains how herbal formulae achieve coordinated therapeutic effects by simultaneously modulating gut ecology, hepatic metabolism, and tissue-specific receptor responses, providing a scientific foundation for next-generation microbiome-informed therapeutics targeting complex chronic diseases.

## 3. Cross-Axis Convergence: Microbiota-BA Hub as Universal Therapeutic Target

The preceding sections have systematically detailed how specific TCM formulations achieve therapeutic benefits across distinct gut–organ axes by modulating the microbiota–BA–host receptor network (Section 2.1, Section 2.2, Section 2.3, Section 2.4 and Section 2.5). To provide a comprehensive and comparative overview of these multi-targeted interventions, key representative formulae and their core mechanistic features are summarized in Table 1 and Table 2. This integrated presentation highlights both the shared therapeutic strategies and the context-specific pathway engagements that will be further examined in the following cross-axis synthesis. The herbal composition and preparation method for each formula are provided in Table 3.

### 3.1. The Pathogenic Triad: How Gut Disruption Drives Multi-Organ Disease

Across all gut–organ axes, disease pathogenesis is consistently initiated by a triad of interconnected disturbances: (1) gut microbial dysbiosis, (2) compromised intestinal barrier integrity (“leaky gut”), and (3) altered production and signaling of gut-derived metabolites, particularly BAs. For example, in the gut–heart axis, dysbiosis and barrier leakage promote translocation of LPS and TMAO, driving vascular inflammation and atherosclerosis. Similarly, in the gut–kidney axis, dysbiosis leads to accumulation of uremic toxins (e.g., IS) and BA dysregulation, exacerbating renal fibrosis. In the gut–liver axis, barrier dysfunction allows endotoxin influx, triggering hepatic inflammation and steatosis. This recurrent pattern underscores that regardless of the distant organ involved, the gut serves as a common pathogenic epicenter, with dysbiosis and barrier breakdown acting as upstream drivers of multi-organ pathology.

### 3.2. Three Core Strategies: Microbial Restructuring, Barrier Repair, BA Reprogramming

In response to these shared pathogenic mechanisms, TCM formulations employ a remarkably consistent mechanistic strategy across different axes, focusing on three core restorative approaches.

#### 3.2.1. Restructuring Gut Microbial Ecology

TCM interventions systematically reshape microbial composition toward a symbiotic state. This commonly involves normalizing the *F*/*B* ratio, enriching beneficial genera (e.g., *Akkermansia*, *Lactobacillus*, *Bifidobacterium*), and suppressing pathobionts (e.g., *Escherichia-Shigella*, *Enterobacteriaceae*). Whether improving cardiac function (e.g., ZXYF) [45], protecting renal tissue (e.g., Yi-Shen-Hua-Shi granules) [77], or alleviating liver steatosis (e.g., LG) [110], the recalibration of microbial community structure is a universal first step.

#### 3.2.2. Reinforcing Intestinal Barrier Integrity

A direct and consistent action across TCM formulations is the upregulation of TJ proteins (e.g., ZO-1, Occludin, Claudins) and the enhancement of mucosal defense. This barrier-fortifying effect is crucial for limiting the systemic translocation of pro-inflammatory agents (LPS) and harmful metabolites. Examples include NXT [50] in atherosclerosis, Shenshuaikang enema [83] in kidney disease, and GQD [115] in metabolic disorders, all demonstrating reduced permeability and endotoxemia.

#### 3.2.3. Modulating the BA Metabolome and Signaling Network

The most profound mechanistic integration occurs at the level of BA metabolism. Across all axes, TCM exerts its effects by strategically modulating the BA pool. This often involves reducing cytotoxic secondary BAs (e.g., DCA, LCA) or conjugated species, while promoting beneficial BA profiles. Subsequently, these modified BAs engage key receptors (FXR, TGR5, PXR, VDR) in a tissue- and context-specific manner to resolve inflammation, mitigate fibrosis, and restore metabolic homeostasis. This BA-centric signaling network serves as the principal mechanistic bridge connecting microbial remodeling to therapeutic outcomes in distant organs.

### 3.3. Plasticity and Context-Dependency: BAs as Central Integrators of Gut–Organ Communication

**Figure 5:**
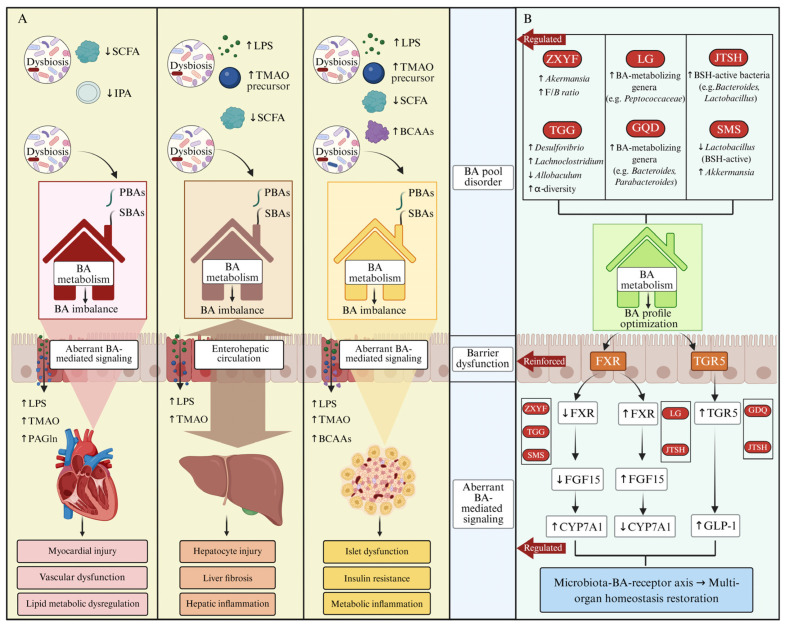
The microbiota-BA axis as a common pathogenic foundation and convergent therapeutic target in extraintestinal diseases. (**A**) BAs function as the key integrator within the gut–X axis framework, bridging gut microbial ecology and systemic pathophysiology across multiple organ axes. Gut dysbiosis and intestinal barrier dysfunction converge to disrupt BA homeostasis, leading to a disordered BA pool, which in turn compromises BA-mediated signaling and ultimately affects the target organ; (**B**) Convergent therapeutic strategies of TCM targeting the microbiota-BA-receptor axis. ↑, increase; ↓, decrease. Arrows indicate direction of effect. Created with BioRender (https://biorender.com/). Abbreviations: BA, bile acid.

The comparative analysis across gut–organ axes establishes BAs as the key integrator within the gut–X axis framework (Figure 5A). This central role is enabled by two fundamental properties: microbiota-dependent plasticity and context-dependent receptor signaling (Figure 5B). Two fundamental properties support this pivotal role. First, BAs exhibit microbiota-dependent plasticity. Their structural modification by gut microbial enzymes, primarily BSH, creates a direct functional link between intestinal microbial ecology and the signaling characteristics of the host BA pool. By reshaping these microbial communities, TCM formulations achieve precise control over the biosynthesis of specific BA species, which subsequently act as targeted agonists or antagonists for host receptors. Second, BA receptor signaling demonstrates pronounced context-dependency. The activation of a single receptor, such as the FXR, can mediate different and sometimes opposing therapeutic outcomes depending on the disease context. This is exemplified by the differential FXR modulation observed with specific TCM formulations: the ZXYF enhanced hepatic FXR activity to promote cholesterol clearance in atherosclerosis [45], whereas TGG suppressed FXR signaling to ameliorate inflammation-mediated hypertension [46]. This inherent flexibility at the receptor level enables TCM to generate precise, pathology-adapted effects through a shared molecular hub.

These converging insights support a systems-level understanding of TCM pharmacology. The therapeutic efficacy of TCM arises not from a collection of isolated organ-specific actions, but from a coordinated capacity to address common upstream pathological drivers, specifically gut dysbiosis and barrier dysfunction, which are fundamental to multiple chronic diseases. The microbiota–BA–host receptor axis operates as the core regulatory circuit in this approach. This circuit effectively translates interventions directed at the gut, such as microbial restructuring and barrier reinforcement, into synchronized benefits across disparate organ systems. Therapeutic specificity is achieved not by deploying entirely different mechanisms for each disease, but through context-dependent fine-tuning of this central circuit, as illustrated by the differential FXR modulation.

### 3.4. Evidence Stratification: Microbiota Dependence Versus BA-Mediated Causality

Building upon the concept of a shared core regulatory circuit, the question of causality requires careful stratification of evidence.

#### 3.4.1. Microbiota Dependence

Antibiotic depletion studies establish that an intact GM is required for TCM efficacy. For instance, the anti-fibrotic effect of BGN was significantly reduced when the microbiota was depleted, providing strong evidence that its efficacy was not merely a direct pharmacological action on the liver [133]. Similarly, FMT experiments demonstrate sufficiency: transferring microbiota from TCM-treated donors (BGN [133], Fuzi [159], or SMS [187]) to diseased recipients reproduced key therapeutic benefits without administering the herbal formula itself. These findings confirm that TCM’s effects are microbiota-dependent but do not identify which microbial metabolites mediate the benefits.

#### 3.4.2. BA Metabolic Correlation

Multi-omics analyses reveal consistent associations between TCM-induced BA profile changes and therapeutic outcomes. Different TCM formulations modulate distinct bacterial groups (e.g., BGN enriches BSH-active *Lactobacillus* [133]; SMS reduces its abundance [187]), leading to context-specific BA alterations (elevated UDCA versus TCA). These BA shifts correlate with the activation of tailored receptor pathways (PPARα in hepatocytes, TGR5 in joints, or neuronal ROBO1) and with disease resolution. Such correlative evidence links BA metabolism to TCM efficacy but does not prove that BAs are the necessary mechanistic intermediary.

#### 3.4.3. BA-Specific Causal Mediation

Direct evidence for BA mediation requires experimental models where BA receptors are genetically or pharmacologically manipulated (e.g., *FXR* or *TGR5* knockout) or where specific BA species are supplemented in germ-free recipients. Currently, across all gut–X axes discussed in this review, no study has provided such direct evidence. The available data, including the SMS study [187], establish microbiota dependence and BA correlation but do not demonstrate that BAs are the necessary and sufficient mediators. Future research should prioritize receptor-specific knockouts and BA reconstitution experiments to elevate correlative findings to causal claims.

The available evidence therefore establishes microbiota dependence and BA metabolic correlation but stops short of demonstrating direct BA-mediated causality. Several unresolved contradictions and questions that arise from this evidence are discussed below in Table 4.

### 3.5. Critical Appraisal of Contradictions and Unresolved Questions

#### 3.5.1. Opposite Modulation of FXR Across Diseases

ZXYF upregulates hepatic FXR in atherosclerosis [45], whereas TGG suppresses FXR in hypertension [46]; both outcomes are reported as beneficial. Whether this opposite modulation reflects genuine disease-specific requirements, differences in dosage, formula composition, or experimental conditions has not been tested in a comparative design. Without head-to-head comparisons under standardized conditions, the claim that FXR serves as a universal therapeutic target in TCM pharmacology remains provisional.

#### 3.5.2. Opposing Roles of the Same BA Molecule Across Axes

TCA illustrates the context-dependent nature of BA signaling. TCA is reduced by JTSH in diabetes [162] but elevated by SMS in obesity [187]; in the gut–liver axis, TCA is considered hepatotoxic and is lowered by YZH and DCHD [111,132]. This suggests that simple classification of BAs as beneficial or detrimental is insufficient; concentration thresholds and tissue-specific responses likely determine net effects. Systematic dose–response studies across disease models are needed to clarify these context-dependent roles.

#### 3.5.3. Direct Receptor Agonism Versus Microbiota-Mediated Effects

Several TCM constituents, including alisol from ZXYF, are established FXR/TGR5 ligands in vitro [45]. Most in vivo studies attribute therapeutic effects to BA modulation without ruling out direct compound-receptor binding. Pharmacokinetic studies measuring tissue concentrations of both BA species and TCM-derived small molecules are needed to dissect these two mechanisms.

## 4. Conclusions

In summary, this review suggests the microbiota–BA–organ axis as a potential framework for understanding the systemic therapeutic actions of TCM. Across the gut–heart, gut–kidney, gut–liver, gut–bone, and gut–endocrine axes, representative TCM formulations appear to remodel gut microbial ecology, reinforce intestinal barrier integrity, and modulate BA profiles, thereby engaging tissue-specific receptors (FXR, TGR5, PXR, VDR) to help resolve inflammation, mitigate fibrosis, and restore metabolic homeostasis. The available evidence, including antibiotic depletion, FMT, and multi-omics profiling, is consistent with a causal and at least partially BA-mediated relationship.

Several limitations should be considered. Most mechanistic evidence comes from rodent models (*db*/*db* mice, HFD-fed mice, STZ-induced rats). While these models are valuable, notable species differences in BA conjugation (glycine in humans, taurine in mice, unconjugated in rats), sulfation versus hydroxylation, MCA production in rodents, and gut microbial transformation capacities suggest caution when extrapolating to humans [190]. These differences do not invalidate rodent studies but define the boundaries for mechanistic inferences. When a mechanism is conserved or supported by human genetic/organoid data, rodent findings retain predictive value; where discrepancies exist, cross-validation using humanized models or clinical cohorts is needed. Furthermore, most studies employed relatively short treatment durations, and long-term safety data as well as independent replication in larger cohorts are generally lacking. Clinical validation of TCM-mediated BA modulation therefore remains an important direction for future investigation. Future studies would benefit from systematically accounting for potential confounding factors such as diet, baseline microbiota composition, and host genetics, and establishing dose–response relationships and pharmacokinetic profiles for TCM-derived BA-modulating compounds also represents an important direction for future investigation. Another consideration is the chemical complexity of TCM formulations. Many were tested as non-standardized extracts, and batch-to-batch variability, lack of identified active compounds for most formulae, and difficulty distinguishing whole-formula effects from individual constituents remain challenges. Furthermore, systematic evaluation of the potential adverse effects of BA modulation, including excessive FXR activation in liver disease and the carcinogenic risks of sustained secondary BA accumulation, remains largely absent in current TCM research and should be prioritized in future studies. This does not invalidate the observed effects but calls for caution in attributing outcomes to specific pathways.

Future priorities include patient stratification based on microbial and BA signatures, development of human-relevant preclinical models (humanized mice, organoids), rigorous clinical trials, bioactivity-guided fractionation, chemical standardization (e.g., chromatographic fingerprinting), and systematic comparisons between crude extracts and purified components. In parallel, the isolation and characterization of TCM derived active compounds with known BA modulating effects, such as alisol, a hepatic FXR agonist from ZXYF [45], and berberine, could facilitate the development of standardized, mechanism based interventions suitable for broader clinical application. Likewise, specific beneficial bacteria enriched by TCM treatments, including *Akkermansia* and *Lactobacillus* strains, may warrant evaluation as standalone probiotic candidates, subject to rigorous strain characterization and clinical testing. The practical adoption of these strategies outside China will require addressing regulatory complexity, ensuring batch to batch consistency through chemical standardization, and generating clinical evidence in diverse populations. As a step toward individualized therapy, BA profiling could assist in matching specific TCM formulas to patient needs; for example, patients with diabetic kidney disease who exhibit elevated serum secondary BAs might be candidates for QDTS, which selectively reduces T-β-MCA and DCA. Notably, early clinical examples lend support to these directions: BSH-active *Lactiplantibacillus plantarum* strains reduced conjugated BAs and improved lipoprotein profiles in overweight adults [191]; a synbiotic formulation of *Bifidobacterium animalis* subsp. *lactis* MN-Gup plus galactooligosaccharide regulated serum BAs and GLP-1 secretion in patients with T2DM [192]; a multi-strain probiotic intervention increased circulating ursodeoxycholate in T2DM patients [193]; and a combination of UDCA with *Bifidobacterium* improved antiviral immunity in patients with chronic hepatitis B [194]. These studies illustrate the translational potential of microbiome-targeted interventions and support further development of engineered probiotics and postbiotic formulations. With respect to TCM formula-based interventions, a pilot observational study reported that YH1, a concentrated herbal extract formula containing Rhizoma *coptidis* and Shen-Ling-Bai-Zhu-San, increased stool tauro-conjugated BA levels and plasma total BAs while reducing unconjugated secondary BAs in male patients with T2DM [195]. Further clinical studies with larger sample sizes and BA-specific endpoints are warranted to extend these early findings. Collectively, these efforts will help move the field from phenomenological observations toward mechanism-based pharmacology and ultimately inform microbiome-targeted, multi-component precision therapeutics.

## Figures and Tables

**Table 1:** Study design and outcomes of TCM interventions targeting the BA–gut–X axis.

Axis	TCM Intervention	Disease	Experimental Model	Key Outcomes	Evidence for BA Mediation	Level of Evidence	References
Gut–heart	ZeXieYin Formula (ZXYF)	Atherosclerosis	Male *ApoE*^−^/^−^ mice + HFD ZXYF 3.25/6.50 g/kg/d or atorvastatin 10 mg/kg/d, oral, 8 w	↓ TC, TG, LDL-C ↑ HDL-C ↓ IL-1β, TNF-α, LPS ↓ Intestinal permeability ↓ Aortic plaque area	/	Animal model	[45]
Gut–heart	Tianma-Gouteng Granules (TGG)	Hypertension	Male SHR + Fuzi decoction TGG 1.5/3.0 g/kg/d or irbesartan 14.7 mg/kg/d, oral, 4 w	↓ SBP, DBP, MAP ↓ IL-1β, IL-6, TNF-α ↓ Renin, AngII	/	Animal model	[46]
Gut–kidney	QiDiTangShen Granules (QDTS)	Diabetic nephropathy	Male *db*/*db* mice QDTS 3.37 g/kg/d or valsartan 10.29 mg/kg/d, oral, 12 w	↓ UAE, KIM-1 ↓ Glomerular hypertrophy ↓ Mesangial expansion ↓ Tubular injury	/	Animal model	[80]
Gut–liver	Ling-Gui-Zhu-Gan Decoction (LG)	NAFLD	Male C57BL/6 mice + HFD LG 715 mg/kg/d, oral, 8 w	↓ Body weight ↓ Liver TG, TC, lipid droplets ↑ Insulin sensitivity ↓ ALT, AST, MDA, LBP	/	Animal model	[110]
Gut–liver	Yinzhihuang Formula (YZH)	Cholestatic liver injury	Male C57BL/6 mice + ANIT (62.5 mg/kg i.p.) YZH 1.35–5.4 g/kg/d or UDCA, oral, 2 w	↓ ALT, ALP ↓ Liver inflammation, fibrosis ↑ Gut barrier ↑ Fecal BA excretion	FMT (yes)	Animal model	[111]
Gut–liver	SanWei GanJiang Powder (SWGJ)	Intestinal dysbiosis & chronic liver injury	Model 1: BALB/c mice (1:1), ceftriaxone 12 g/kg/d, oral, 10 d; SWGJ 0.33/0.66 g/kg/d or Bifico, 7 d.	↓ Ileal inflammation, LPS ↓ Serum ALT, AST ↑ ZO-1, Occludin ↑ Hepatic Nrf2	/	Animal model	[112]
			Model 2: SD rats (1:1), CCl_4_ 40% s.c., 8 w; SWGJ 0.165/0.66 g/kg/d or Silymarin, 6 w				
Gut–liver	Da-Chai-Hu Decoction (DCHD)	ANIT- and BDL-induced cholestasis	Male C57BL/6J mice; ANIT i.p. or BDL surgery DCHD 6–24 g/kg/d (ANIT) or 12–48 g/kg/d (BDL), oral, 15–20 d	↓ ALT, AST, ALP, TBA, TBIL ↓ Hepatic necrosis, inflammation, fibrosis ↓ Systemic/hepatic NF-κB	/	Animal model	[132]
Gut–liver	Bao-Gan-Ning Decoction (BGN)	Hepatic fibrosis	Male C57BL/6 mice + CCl_4_ BGN 12.74–50.96 g/kg/d or Anluohuxian 1.82 g/kg/d, oral, 6 w	↓ ALT, AST, collagen, α-SMA Restored gut barrier integrity	ABX (yes) FMT (yes)	Animal model	[133]
Gut–liver	Danggui Shaoyao San (DSS)	Hepatic fibrosis	Male SD rats + CCl_4_ DSS 2–8 g/kg/d or silymarin 50 mg/kg/d, oral, 8 w	↓ Collagen, Ishak score ↑ Thymic index ↓ LPS, D-lactate ↑ Fecal SCFAs Improved barrier	/	Animal model	[113]
Gut–liver	Gegen-Qinlian Decoction (GQD)	MASH	Male C57BL/6J mice + FPC diet GQD 3–12 g/kg/d, oral, 4 w	↓ Liver TG, cholesterol, steatosis ↓ ALT, ALP ↓ Body weight, TC, LDL-C, LPS ↑ Gut barrier integrity	/	Animal model	[115]
Gut–joint	Fuzi	Cold-related RA	Male Wistar rats, CIA + cold exposure Fuzi extract 0.625 g/mL or methotrexate, oral, d16-32	↓ Arthritis index, paw swelling ↑ Bone parameters (BMD, BV/TV) ↑ Energy metabolism hormones	FMT (yes)	Animal model	[159]
Gut–endocrine	Jiang-Tang-San-Huang Pill (JTSH)	T2DM	Male SD rats, HFD + STZ JTSH 0.27–1.08 g/kg/d or metformin 0.2 g/kg/d, oral, 4 w	↓ Fasting glucose, HOMA-IR ↓ Lipids ↓ Inflammation Improved ileal integrity	/	Animal model	[162]
Gut–endocrine	Shengmai San (SMS)	Obesity	Male C57BL/6J mice + HFD SMS 2–8 g/kg/d or orlistat 20 mg/kg/d, oral, 6 w	↓ Body weight, hepatic/serum lipids ↓ Glucose ↑ Energy expenditure ↑ Adipose browning	ABX (yes) FMT (yes)	Animal model	[187]

Note: ↑, increase; ↓, decrease. Abbreviations: w, weeks; d, day(s); ABX, antibiotics; ALP, alkaline phosphatase; ALT, alanine aminotransferase; AngII, angiotensin II; AST, aspartate aminotransferase; BMD, bone mineral density; BV/TV, bone volume/total volume; CIA, collagen-induced arthritis; DBP, diastolic blood pressure; FMT, fecal microbiota transplantation; FPC, fructose-palmitate-cholesterol diet; HDL-C, high-density lipoprotein cholesterol; HFD, high-fat diet; HOMA-IR, homeostatic model assessment of insulin resistance; IL-1β, interleukin-1β; IL-6, interleukin-6; KIM-1, kidney injury molecule-1; LDL-C, low-density lipoprotein cholesterol; LPS, lipopolysaccharide; MAP, mean arterial pressure; MDA, malondialdehyde; NAFLD, non-alcoholic fatty liver disease; RA, rheumatoid arthritis; SBP, systolic blood pressure; SCFAs, short-chain fatty acids; STZ, streptozotocin; T2DM, type 2 diabetes mellitus; TBA, total bile acids; TBIL, total bilirubin; TC, total cholesterol; TG, triglycerides; TNF-α, tumor necrosis factor-α; UAE, urinary albumin excretion; UDCA, ursodeoxycholic acid; ZO-1, zonula occludens-1.

**Table 2:** Mechanistic data: GM, BA alterations and signaling pathways.

Axis	TCM Intervention	GM Changes	BA Alterations	Molecular Signaling Changes	Core Mechanistic Pathway	References
Gut–heart	ZXYF	↑ *Akkermansia* ↓ *F*/*B* ratio	↓ Fecal DCA, LCA ↑ Fecal TBA, cholesterol	↓ Ileal FXR, FGF15 ↑ Hepatic CYP7A1, ABCG5/8	Enrich *Akkermansia* → reduce DCA/LCA → inhibit ileal FXR/FGF15 → upregulate hepatic CYP7A1/ABCG5/8 → enhance cholesterol/BA excretion → repair gut barrier	[45]
Gut–heart	TGG	↑ *Desulfovibrio*, *Lachnoclostridium* ↓ *Allobaculum* ↑ α-diversity	↑ Serum/intestine TBA ↓ Hepatic TBA	↓ Ileal/liver FXR, FGF15 ↑ Hepatic CYP7A1	Modulate microbiota (↑ *Desulfovibrio*) → inhibit ileal FXR/FGF15 → activate hepatic CYP7A1 → reshape BA pool → reduce inflammation → lower BP	[46]
Gut–kidney	QDTS	↑ *Alloprevotella* ↓ *Lachnospiraceae_NK4A136_group*, *Lactobacillus*	↓ Serum TBA, β-MCA, TCA, DCA ↑ FXR agonist/antagonist ratio	/	Remodel microbiota (↑ SCFA producers) → reduce Tβ-MCA, DCA → alleviate glomerular/tubular injury	[80]
Gut–liver	LG	↓ *Proteobacteria* ↑ SCFAs	↓ Serum DCA, HDCA	↑ Ileal FGF15, FXR, Occludin, ZO-1 ↓ Hepatic CYP7A1, PPARγ, inflammatory genes	Rebalance microbiota → ↑ SCFAs, ↓ secondary BAs → enhance barrier & ileal FXR/FGF15 → downregulate hepatic CYP7A1/PPARγ → ameliorate NAFLD	[110]
Gut–liver	YZH	↑ *Clostridiales*, *Lachnospiraceae* ↓ *Escherichia-Shigella*	↓ Serum TCA, T-β-MCA ↑ Fecal BSH activity	↓ Hepatic CYP7A1	Repair barrier & remodel microbiota → reduce bacterial translocation & toxic BA absorption → promote fecal BA excretion → attenuate cholestasis	[111]
Gut–liver	SWGJ	Restored *F*/*B* ratio ↑ *Lactobacillus*	Not directly measured	↑ Hepatic CYP7A1, NTCP, BSEP ↑ Nuclear Nrf2 ↓ Bach1	Restore microbiota & barrier → reduce endotoxemia → activate hepatic Nrf2 → upregulate BA transporters → normalize BA circulation	[112]
Gut–liver	DCHD	Not directly measured	↓ Toxic/pro-inflammatory BAs (TCA, TMCA) ↑ Protective BAs (TUDCA, THDCA)	↑ Hepatic FXR, BSEP	Activate hepatic FXR → upregulate BSEP/MRP2 → enhance BA excretion → inhibit classical BA synthesis → shift to alternative pathway → reduce toxic BAs → alleviate cholestasis	[132]
Gut–liver	BGN	↑ *Lactobacillus*, *Bifidobacterium* ↓ Pathogens ↑ Microbial diversity	↑ UDCA ↓ TCA, TMCA	↑ BA transporters ↑ PPARα, CYP7A1	Enrich probiotics → enhance BSH activity → promote BA deconjugation → activate PPARα/CYP7A1 → upregulate BA transporters → restore BA homeostasis & barrier → attenuate fibrosis	[133]
Gut–liver	DSS	↑ SCFA-producing & BA-metabolizing genera Restored *F*/*B* ratio	↓ Toxic BAs (CA, TCA) ↑ Fecal BA excretion ↑ BSH activity	↑ Hepatic CYP7A1, BSEP	Enrich SCFA/BA-metabolizing bacteria → increase SCFAs & reshape BA profile → enhance barrier & reduce endotoxemia → upregulate hepatic BA excretion → inhibit HSC activation → alleviate fibrosis	[113]
Gut–liver	GQD	↑ *Akkermansia* ↓ *Desulfovibrio*, *Helicobacter*	↓ Fecal conjugated BAs ↑ Sulfated BAs	↑ Intestinal TGR5, VDR	Modulate microbiota → reshape BA profile → activate intestinal TGR5/VDR → enhance gut barrier → reduce systemic inflammation → ameliorate MASH	[115]
Gut–joint	Fuzi	↑ *Lachnospiraceae*, *Ruminococcaceae* ↓ *Eggerthellaceae*	↑ Fecal/serum TCA, THDCA	↑ TGR5, cAMP, PKA ↓ NLRP3	Remodel microbiota → increase THDCA → activate TGR5-cAMP-PKA → inhibit NLRP3 inflammasome → reduce synovial inflammation & bone damage	[159]
Gut–endocrine	JTSH	↑ BSH-active bacteria (*Bacteroides*, *Lactobacillus*)	↓ TCA ↑ Unconjugated CDCA, DCA	↑ Ileal FXR, FGF15, TGR5, GLP-1 ↓ Hepatic CYP7A1, CYP8B1	Enrich BSH-active microbiota → increase unconjugated BAs → activate intestinal FXR & TGR5/GLP-1 → inhibit hepatic BA synthesis → enhance GLP-1 & insulin sensitivity	[162]
Gut–endocrine	SMS	↓ *Lactobacillus* (BSH-active) ↑ *Akkermansia*	↑ T-conjugated BAs (TCA) ↓ Ileal BSH activity	↓ Ileal FXR, FGF15 ↑ Hepatic CYP7A1 ↑ iWAT: M2 macrophages, Slit3, PKA/CaMKII, NE, pHSL, UCP1	Modulate microbiota (↓ BSH) → increase TCA → suppress ileal FXR/FGF15 → promote hepatic BA synthesis → TCA in iWAT induces M2 polarization & Slit3 → activates sympathetic neurons → enhances lipolysis & browning	[187]

Note: ↑, increase; ↓, decrease. Abbreviations: ABCG5/8, ATP-binding cassette subfamily G members 5/8; BP, blood pressure; BSEP, bile salt export pump; BSH, bile salt hydrolase; CaMKII, calcium/calmodulin-dependent protein kinase II; cAMP, cyclic adenosine monophosphate; CDCA, chenodeoxycholic acid; CYP7A1, cholesterol 7α-hydroxylase; CYP8B1, sterol 12α-hydroxylase; DCA, deoxycholic acid; *F*/*B*, *Firmicutes*/*Bacteroidetes* ratio; FGF15, fibroblast growth factor 15; FXR, farnesoid X receptor; GLP-1, glucagon-like peptide-1; HDCA, hyodeoxycholic acid; HSC, hepatic stellate cell; iWAT, inguinal white adipose tissue; LCA, lithocholic acid; MCA, muricholic acid; MRP2, multidrug resistance-associated protein 2; NE, norepinephrine; NLRP3, NLR family pyrin domain containing 3; Nrf2, nuclear factor erythroid 2-related factor 2; NTCP, sodium/taurocholate cotransporting polypeptide; OSTα/β, organic solute transporter alpha/beta; PKA, protein kinase A; PPARα, peroxisome proliferator-activated receptor alpha; PPARγ, peroxisome proliferator-activated receptor gamma; pHSL, phospho-hormone-sensitive lipase; SCFAs, short-chain fatty acids; TBA, total bile acids; TCA, taurocholic acid; TGR5, Takeda G protein-coupled receptor 5; THDCA, taurohyodeoxycholic acid; Tβ-MCA, tauro-β-muricholic acid; TUDCA, tauroursodeoxycholic acid; UCP1, uncoupling protein 1; UDCA, ursodeoxycholic acid; VDR, vitamin D receptor; ZO-1, zonula occludens-1.

**Table 3:** Herbal composition and preparation methods of the TCM formulas.

Formula	Herbal Ingredients (Botanical Names/Chinese Names)	Preparation Method	Reference
ZXYF	*Alisma plantago-aquatica* L. (Zexie), *Atractylodes macrocephala* Koidz. (Baizhu), *Pyrola calliantha* H. Andres. (Luxinacao)	Decoction	[45]
TGG	*Gastrodia elata* Blume (Tianma), *Uncaria rhynchophylla* (Miq.) Miq. ex Havil. (Gouteng), *Haliotis diversicolor* Reeve (Shijueming), *Gardenia jasminoides* Ellis (Zhizi), *Scutellaria baicalensis* Georgi (Huangqin), *Achyranthes bidentata* Blume (Niuxi), *Eucommia ulmoides* Oliv. (Duzhong), *Leonurus japonicus* Houtt. (Yimucao), *Taxillus chinensis* (DC.) Danser (Sangjisheng), *Poria cocos* (Schw.) Wolf. (Fuling), *Polygonum multiflorum* Thunb. (Shouwuteng)	Granule	[46]
QDTS	*Rehmannia glutinosa* (Gaertn.) DC. (Dihuang), *Astragalus propinquus* Schischkin (Huangqi), *Euryale ferox* Salisb. (Qianshi), *Cornus officinalis* Sieb. & Zucc. (Shanzhuyu), *Whitmania pigra* Whitman (Shuizhi), *Rheum officinale* Baill. (Dahuang), *Hedyotis diffusa* Willd. (Baihuasheshecao)	Granule (water extract)	[80]
LG	*Poria cocos* (Schw.) Wolf. (Fuling), *Cinnamomum cassia* Presl (Guizhi), *Atractylodes macrocephala* Koidz. (Baizhu), *Glycyrrhiza uralensis* Fisch. (Gancao)	Decoction	[110]
YZH	*Artemisia capillaris* Thunb. (Yinchen), *Gardenia jasminoides* Ellis (Zhizi), *Scutellaria baicalensis* Georgi (Huangqin), *Lonicera japonica* Thunb. (Jinyinhua)	Oral liquid/decoction	[111]
SWGJ	*Zingiber officinale* Roscoe (Ganjiang), *Alpinia katsumadai* Hayata (Doukou), *Myristica fragrans* Houtt. (Roudoukou)	Powder (suspended in water)	[112]
DCHD	*Bupleurum falcatum* L. (Chaihu), *Scutellaria baicalensis* Georgi (Huangqin), *Paeonia lactiflora* Pall. (Baishao), *Pinellia ternata* (Thunb.) Makino (Banxia), *Citrus × aurantium* L. (Zhishi), *Rheum palmatum* L. (Dahuang), *Zingiber officinale* Roscoe (Shengjiang), *Ziziphus jujuba* Mill. (Dazao)	Fluid extract/decoction	[132]
BGN	*Mallotus apelta* (Lour.) Mull.Arg. (Baibeiyegen), *Eupolyphaga sinensis* Walker (Tubiechong), *Curcuma phaeocaulis* Valeton (Ezhu), *Panax notoginseng* (Burkill) F.H. Chen (Sanqi), *Astragalus aaronii* (Eig) Zohary (Huangqi), *Salvia miltiorrhiza* Bunge (Danshen), *Alchornea trewioides* (Benth.) Mull.Arg. (Hongbeiyegen)	Decoction	[133]
DSS	*Angelica sinensis* (Oliv.) Diels (Danggui), *Poria cocos* (Schw.) Wolf. (Fuling), *Alisma orientale* (Sam.) Juzep. (Zexie), *Ligusticum chuanxiong* Hort. (Chuanxiong), *Atractylodes macrocephala* Koidz. (Baizhu), *Paeonia lactiflora* Pall. (Baishao)	Decoction (50% ethanol extract, lyophilized)	[113]
GQD	*Pueraria lobata* (Willd.) Ohwi (Gegen), *Scutellaria baicalensis* Georgi (Huangqin), *Coptis chinensis* French (Huanglian), *Glycyrrhiza uralensis* Fisch. (Gancao)	Granule extract (reconstituted in water)	[115]
Fuzi	*Aconitum carmichaelii* Debx. (Fuzi)	Decoction (prolonged boiling for detoxification)	[159]
JTSH	*Astragalus mongholicus* Bunge (Huangqi), *Prunus persica* (L.) Batsch (Taoren), *Rheum officinale* Baill. (Dahuang), *Rehmannia glutinosa* (Gaertn.) DC. (Dihuang), *Ophiopogon japonicus* (Thunb.) Ker Gawl. (Maidong), *Scrophularia ningpoensis* Hemsl. (Xuanshen), *Cinnamomum cassia* (L.) J.Presl (Rougui), *Glycyrrhiza uralensis* Fisch. (Gancao)	Pill (water soluble, suspended in water)	[162]
SMS	*Panax ginseng* C.A. Mey. (red ginseng, Hongshen), *Ophiopogon japonicus* (Thunb.) Ker Gawl. (Maidong), *Schisandra chinensis* (Turcz.) Baill. (Wuweizi)	Ethanol extract (percolation), lyophilized powder	[187]

**Table 4:** Critical appraisal of contradictions and suggested experiments.

Contradiction or Gap	Relevant TCM Formulas	Current Evidence Status	Suggested Experiment
Opposite FXR modulation (ZXYF activates, TGG suppresses) is beneficial in different diseases	ZXYF [45] TGG [46]	BA correlation: established for both Microbiota dependence: untested FXR causality: untested	Co-administer FXR agonist/antagonist in each disease model Assess whether reinforcing or blocking FXR alters outcome Determine if effect is disease-specific or formula-dependent
TCA is elevated in obesity (SMS) but reduced in diabetes (JTSH) and cholestasis (YZH, DCHD)	SMS [187] JTSH [162] YZH [111] DCHD [132]	BA correlation: established for all four SMS microbiota dependence: confirmed (ABX/FMT) JTSH, YZH, DCHD: untested	Dose–response studies of TCA across metabolic and cholestatic models Identify concentration thresholds and tissue-specific effects
In vitro FXR/TGR5 ligands (e.g., alisol) are present, but in vivo effects are attributed to BA modulation	ZXYF [45]	BA correlation: established Direct receptor binding: confirmed in vitro Relative contribution: unresolved	Compare BA profiles and FXR activation in ABX-depleted vs. microbiota-intact mice given ZXYF Measure tissue alisol and BA levels simultaneously
Microbiota dependence (FMT/ABX) is confirmed in some studies, but direct BA causality (receptor KO or BA reconstitution) is not tested	BGN [133] Fuzi [159] SMS [187]	BGN: ABX/FMT confirmed Fuzi: FMT confirmed SMS: ABX/FMT confirmed Direct BA causality: untested	FMT from TCM-treated donors into germ-free recipients with FXR/TGR5 knockout Test with/without BA reconstitution to establish causality

## Data Availability

No new data were created or analyzed in this study.
